# CCL3^+^ Neutrophil Signature Predicts Response to Neoadjuvant Toripalimab plus Chemotherapy in Patients with Hypopharyngeal Squamous Cell Carcinoma: A Phase II Trial

**DOI:** 10.1158/1078-0432.CCR-25-4096

**Published:** 2026-03-12

**Authors:** Fang Chen, Shengli Zhou, Juke Ma, Hengmin Tao, Peihang Jing, Xuliang Liu, Zhong Shen, Zhichao Liu, Yumei Wei, Zhenghua Lv, Wei Xu

**Affiliations:** 1 Department of Otolaryngology-Head and Neck Surgery, Shandong Provincial ENT Hospital, Shandong University, Jinan, China.; 2 Department of Head and Neck Radiotherapy, Shandong Provincial ENT Hospital, Shandong University, Jinan, China.; 3 Shandong Provincial Key Medical and Health Laboratory of Theranostic Fluorescent Materials for Head and Neck Cancer, Shandong Institute of Otorhinolaryngology, Jinan, China.

## Abstract

**Purpose:**

Hypopharyngeal squamous cell carcinoma (HPSCC) has a poor prognosis. Although neoadjuvant chemoimmunotherapy (nCIT) is promising, responses are heterogeneous, and the PD-L1 combined positive score (CPS) inadequately stratifies benefit. We sought biomarkers to guide patient selection.

**Patients and Methods:**

In this prospective, single-center, single-arm phase II trial, patients with resectable locally advanced HPSCC received two cycles of neoadjuvant toripalimab, albumin-bound paclitaxel, and nedaplatin. The primary endpoint was the pathologic complete response (pCR) rate. Pretreatment tumor biopsies from a subset of patients (*n* = 13) were analyzed by single-cell RNA sequencing to identify determinants of response. Findings were validated in a larger cohort (*n* = 60) using bulk RNA-seq and immunohistochemistry.

**Results:**

Among 70 evaluable patients, the objective response rate was 82.7%. Of the 64 patients who underwent surgery, the pCR rate was 29.7% (95% confidence interval, 18.9%–42.7%). Baseline PD-L1 CPS was not associated with pathologic response (*P* = 0.313). Single-cell analysis revealed that the pretreatment tumor microenvironment of responders was significantly enriched with a proinflammatory neutrophil subset characterized by high expression of *CCL3* (Neu_CCL3). A gene signature score derived from this subset was a strong and independent predictor of pCR (AUC = 0.788), significantly outperforming PD-L1 CPS (AUC = 0.621).

**Conclusions:**

The efficacy of nCIT in HPSCC is predetermined by a baseline immune architecture orchestrated by a CCL3^+^ neutrophil subset. The Neu_CCL3 gene signature is a promising, clinically translatable biomarker that can fill a critical gap in precision immunotherapy for HPSCC.

**Translational Relevance**

Hypopharyngeal squamous cell carcinoma (HPSCC) carries a dismal prognosis, and patient responses to neoadjuvant chemoimmunotherapy (nCIT) are heterogeneous. The current standard biomarker, PD-L1 combined positive score (CPS), has proven unreliable for patient stratification, creating an urgent clinical need for robust predictive tools. In a prospective phase II trial, we combined clinical outcomes with multiomics profiling to address this gap. We identified a proinflammatory neutrophil subset characterized by high *CCL3* expression (Neu_CCL3) that preconfigures an immune-permissive tumor microenvironment in responders. A gene signature derived from this subset robustly predicted pathologic complete response, significantly outperforming PD-L1 CPS. This work nominates the Neu_CCL3 signature as a promising, clinically translatable candidate biomarker to guide patient selection for nCIT in HPSCC, pending multicenter validation and assay standardization. Furthermore, our mechanistic findings suggest a novel therapeutic strategy: pharmacologically inducing this specific neutrophil program to potentially convert immunologically “cold” nonresponders into “hot” responders.

## Introduction

Hypopharyngeal squamous cell carcinoma (HPSCC) is one of the most aggressive and prognostically challenging subtypes of head and neck cancer. Owing to its anatomically concealed location, HPSCC is often diagnosed at a locally advanced stage, frequently with extensive lymph node metastasis (1, 2). Despite multimodal treatment regimens involving surgery, radiotherapy, and chemotherapy, outcomes for HPSCC remain poor, with survival rates lagging significantly behind those of other head and neck cancers, thus posing a major clinical challenge (3). Consequently, there is an urgent need to develop more effective therapeutic strategies to improve the prognosis for this high-risk population.

Neoadjuvant chemoimmunotherapy (nCIT) has emerged as a promising strategy for locally advanced HPSCC (4). The rationale is that chemotherapy-induced tumor cell death releases a plethora of tumor-associated antigens, enhancing immunogenicity, whereas immune checkpoint inhibitors unleash suppressed T cells, synergizing to activate a potent antitumor immune response (5). This strategy of introducing immunotherapy in the presurgical window aims to leverage intact tumor-draining lymph nodes and a more robust immune system to eliminate micrometastases, reduce recurrence risk, and potentially enable organ preservation (6). Toripalimab, an anti–PD-1 antibody, has demonstrated efficacy and safety across various cancers, leading to its approval for malignancies such as non–small cell lung cancer (NSCLC), melanoma, and nasopharyngeal carcinoma (7). Toripalimab-based neoadjuvant regimens have shown encouraging activity in HNSCC (8). However, dedicated data for toripalimab-based neoadjuvant therapy in HPSCC are lacking, and the benefit remains heterogeneous (9). Moreover, the PD-L1 combined positive score (CPS) has shown limited predictive value, underscoring the need for better biomarkers.

A growing body of evidence suggests that the key to predicting immunotherapy response lies within the pretreatment tumor microenvironment (TME; ref. 10). The TME is a complex ecosystem of tumor cells, immune cells, stromal cells, and various cytokines, in which composition and functional state collectively determine the tumor’s immune phenotype (11, 12). Among the diverse cell types in the TME, neutrophils are one of the most abundant and exhibit remarkable functional plasticity (13). Neutrophils have traditionally been considered short-lived, anti-infective proinflammatory cells that directly kill tumor cells via degranulation and cytotoxicity. However, recent studies have found that under the guidance of different microenvironmental signals, neutrophils exhibit heterogeneity and plasticity, and certain subsets can coordinate immune networks to exert anti- or protumor effects (14–16). Whether a critical cellular network—and specifically, whether a unique neutrophil subset—plays a dominant role in determining therapeutic outcomes in the presurgical HPSCC TME remains a key unanswered question.

Here, we combine high-dimensional single-cell RNA sequencing (scRNA-seq) with bulk transcriptomics to systematically resolve the pretreatment TME cellular atlas associated with nCIT efficacy in HPSCC. We show that the TME of responders (R) is preconfigured with a unique, proinflammatory neutrophil subset characterized by the expression of the chemokine *CCL3* (Neu_CCL3). This subset, which is significantly enriched in patients achieving a pathologic complete response (pCR), acts as a central coordinator. Mechanistically, Neu_CCL3 cells secrete a network of chemokines that recruit and activate effector cells, including early-activated CD8^+^ T cells, into the tumor core, thereby sculpting an “immune-permissive” TME. Based on this mechanism, we developed a Neu_CCL3 gene signature score that serves as a robust and independent predictor of pCR, substantially outperforming the PD-L1 CPS and all other clinical features. Our work not only establishes a central coordinating role for neutrophils in HPSCC antitumor immunity but also provides a predictive tool with immediate translational potential, paving the way for precision immunotherapy in this challenging disease.

## Patients and Methods

### Study design and participants

This prospective, single-center, single-arm, exploratory phase II trial was conducted at the Shandong Provincial ENT Hospital, China, and registered with the Chinese Clinical Trial Registry (ChiCTR2400081826; registration date: March 13, 2024). Eligible patients were aged 18 to 75 years with previously untreated, histologically confirmed, resectable, locally advanced HPSCC (clinical stage III–IVA, American Joint Committee on Cancer eighth edition). Key eligibility criteria included an Eastern Cooperative Oncology Group performance status of 0 to 1 and adequate organ function. Major exclusion criteria included prior systemic therapy for HPSCC, distant metastases, autoimmune disease requiring systemic steroids or immunosuppressive drugs, and known contraindications to any study drug. However, all consecutively enrolled participants during the accrual period were male, consistent with the marked male predominance of HPSCC reported in contemporary Chinese cohorts and population-based datasets (17, 18). Therefore, sex-stratified analyses could not be performed in the present study. The representativeness of the study participants is summarized in Supplementary Table S1.

### Ethics statement and trial registration

The study protocol and all its amendments were approved by the Institutional Review Board and Ethics Committee of Shandong Provincial ENT Hospital (No. XYK20210252). The trial was conducted in accordance with the principles of the Declaration of Helsinki and the International Council for Harmonisation of Technical Requirements for Pharmaceuticals for Human Use Good Clinical Practice guidelines. All patients provided written informed consent before the initiation of any study-related procedures, agreeing to participate in the trial, undergo surgical treatment, provide biological samples for research, and allow the publication of anonymized data. The full study protocol is provided as Supplementary File S1.

### Treatment procedures

Patients received two 21-day cycles of neoadjuvant therapy consisting of toripalimab (240 mg on day 1), albumin-bound paclitaxel (125 mg/m^2^ on day 2), and nedaplatin (80 mg/m^2^ on day 2). After completing the two cycles, patients underwent contrast-enhanced computed tomography (CT) of the neck and chest and fiberoptic laryngoscopy to assess response. The surgical plan was determined by a multidisciplinary team, with a preference for larynx-preserving surgery whenever feasible.

### Efficacy assessment

Radiologic tumor response was assessed by two senior radiologists according to the Response Evaluation Criteria in Solid Tumors, version 1.1 (19). The response of the primary hypopharyngeal lesion was evaluated by comparing pre- and posttreatment fiberoptic laryngoscopy findings. The pathologic treatment effect (PTE) was defined as the percentage of the tumor bed area showing tumor necrosis with a histiocytic inflammatory response and/or a multinucleated giant cell reaction to keratin debris. Pathologic response was graded based on PTE as non–major pathologic response (non-MPR; PTE <90%), major pathologic response (MPR; PTE ≥90%), or pCR (PTE = 100%). To facilitate cross-trial interpretability, these PTE cutoffs were selected to conceptually align with commonly used residual viable tumor (RVT)–based definitions in neoadjuvant immunotherapy studies (MPR, ≤10% RVT; pCR, 0% RVT) while acknowledging that scoring systems are not fully harmonized across studies (20). Radiologic response was evaluated by two radiologists blinded to clinical outcomes, and pathologic response was assessed by pathologists blinded to radiologic response and biomarker data. All eligible patients who received protocol therapy were included in the primary efficacy and safety analyses; pathologic response analyses were performed in patients who underwent surgery with available resection specimens.

Perioperative adverse events (AE) were identified and graded according to the National Cancer Institute Common Terminology Criteria for Adverse Events, version 5.0. Event-free survival (EFS) was defined as the time from the start of treatment to tumor recurrence or death from any cause. Overall survival (OS) was defined as the time from the start of treatment to death from any cause.

### Human tissue samples

Pretreatment fresh primary tumor biopsies were collected from patients with locally advanced HPSCC who received nCIT at our institution between March 2023 and July 2024. All enrolled tumors were visible on laryngoscopy and accessible for biopsy. Diagnostic biopsy specimens were stained with hematoxylin and eosin (H&E) and independently reviewed by two experienced pathologists to confirm the presence of tumor tissue. For additional analyses, pretreatment formalin-fixed, paraffin-embedded (FFPE) tumor tissue blocks and associated clinical data were retrospectively collected from patients who underwent nCIT followed by surgery at our institution.

### Molecular profiling and bioinformatics

For scRNA-seq, fresh tumor samples were dissociated into single-cell suspensions using CellLIVE Tissue Dissociation Solution (Singleron Biotechnologies) and diluted to a final concentration of 2.5 to 3.5 × 10^5^ cells/mL. Single-cell suspensions were loaded onto a microwell-based SCOPE-chip (Singleron Biotechnologies), and libraries were generated through cell barcoding, reverse transcription, cDNA amplification, fragmentation, and adapter ligation for Illumina-compatible sequencing. Quality control and downstream analyses were performed in R (version 4.3.1; RRID:SCR_001905) using Seurat (version 5.0.3; RRID:SCR_016341). Low-quality cells were excluded according to the following criteria: detected genes per cell between 300 and 5,000, total unique molecular identifiers (UMI) >1,000 and not exceeding the top 3% of all cells, mitochondrial gene UMI percentage <20%, and red blood cell gene UMI percentage <3%. After normalization and identification of highly variable genes, principal component analysis, shared nearest-neighbor graph construction, clustering, and Uniform Manifold Approximation and Projection (UMAP) visualization were performed. Batch effects between samples were corrected using Harmony (version 1.2.0; RRID:SCR_022206). Cell clusters were annotated using cluster-specific differentially expressed genes (DEG) and canonical marker genes identified by FindAllMarkers.

Intercellular communication was inferred using CellChat (version 1.6.1; RRID:SCR_021946). Copy-number variation profiles were inferred using inferCNV (version 1.18.1; RRID:SCR_021140) with 5,000 T cells and 5,000 B cells as reference normal cells. Functional signature scores were calculated using AddModuleScore in single-cell data and single-sample gene set enrichment analysis (GSEA) in bulk RNA-seq data. Gene expression differences between groups were identified using Seurat-based Wilcoxon rank-sum testing, and GSEA was performed using fgsea (version 1.28.0; RRID:SCR_020938) against Gene Ontology (GO) biological processes, Kyoto Encyclopedia of Genes and Genomes, and Hallmark gene sets from msigdbr (version 7.5.1; RRID:SCR_022870). Differences in cell proportions between groups were assessed using a two-sided unpaired Wilcoxon rank-sum test with Benjamini–Hochberg correction where appropriate.

For bulk RNA-seq, total RNA was extracted from pretreatment fresh tumor biopsies using TRIzol Reagent (Invitrogen). RNA quality was assessed using an Agilent 2200 Bioanalyzer, and samples with an RNA integrity number >7 were used for library construction. cDNA libraries were prepared from 1 μg total RNA using the VAHTS Universal V6 RNA-seq Library Prep Kit for Illumina (Vazyme, Inc.) according to the manufacturer’s instructions and sequenced on a DNBSEQ-T7 platform with 150-bp paired-end reads. Clean reads were aligned to the human reference genome (GRCh38, Ensembl 104) using STAR (version 2.7.10b; RRID:SCR_004463), and gene-level counts were generated using HTSeq-count (version 0.13.5; RRID:SCR_005514). We additionally collected three published cohorts receiving neoadjuvant anti–PD-1 plus chemotherapy from public databases, including GSE207422, GSE91061, and PRJEB23709, as external validation datasets. Expression matrices were harmonized by log_2_(TPM + 1) transformation when required. Receiver operating characteristic analysis and logistic regression were used to evaluate the predictive performance of candidate signatures.

### Tissue microarrays, immunostaining, imaging, and quantification

Tissue microarrays (TMA) were constructed from 56 pretreatment FFPE HPSCC tumor blocks. Tumor-rich regions were identified on H&E-stained sections, and 1-mm cores were extracted and arrayed into recipient paraffin blocks. TMA sections (4 μm) were used for immunohistochemistry (IHC) and multiplex immunofluorescence (IF). For IHC, sections were stained with rabbit anti-human CD15 (Abcam, cat. #ab135377; RRID:AB_3662850) and rabbit anti-human CD68 (Abcam, cat. #ab303565; RRID:AB_3075482) with overnight incubation at 4°C. For IF, sections were stained with antibodies against CD15 (Abcam, cat. #ab241552; RRID:AB_3675595) and CCL3 (Abcam, cat. #ab259372; RRID:AB_3094596), followed by corresponding secondary antibodies; CD15 was visualized in red, and CCL3 was visualized in green, and nuclei were counterstained with DAPI. Whole-slide bright-field images were acquired at 20× magnification using a KFBIO (Konfoong Biotech) scanner, and fluorescence images were acquired on a Leica SP8 laser scanning confocal microscope. Quantification was performed per TMA core using QuPath (RRID:SCR_018257).

### Cell culture

The mouse oral cancer-1 (MOC1, RRID:CVCL_ZD32) and mouse oral cancer-2 (MOC2, RRID:CVCL_ZD33) cell lines were obtained from Qingqi (Shanghai) Biotechnology Development Co., Ltd. in 2023. MOC1 and MOC2 cells were cultured in high-glucose DMEM supplemented with 10% fetal bovine serum at 37°C in a humidified incubator with 5% CO_2_. All cell lines were authenticated by the supplier using short tandem repeat profiling. In our laboratory, all cell lines were tested for mycoplasma contamination after each thawing/recovery. For *in vivo* experiments, all cell lines were passaged at least once after thawing and harvested in the logarithmic growth phase for subcutaneous tumor implantation.

### Animal experiments

All animal experiments were performed in accordance with protocols approved by the Ethics Committee of Shandong Provincial ENT Hospital (No. XYK20210253) and are reported in compliance with the ARRIVE guidelines. Mice were randomly assigned to treatment groups. As this study primarily investigated immune features associated with differential responses to nCIT in patients with HPSCC (all male), sex was not considered a biological variable in the study design, and only male mice were used.

Male C57BL/6J mice (6–8 weeks old; RRID:IMSR_JAX:000664) were purchased from the Animal Center of Shandong University. Mice were acclimated for 7 days and housed under SPF conditions in ventilated cages at 22°C to 24°C with a 12-hour light/12-hour dark cycle, with *ad libitum* access to food and water.

#### Tumor implantation and monitoring

To establish tumor models, single-cell suspensions of MOC1 (5 × 10^6^ cells) or MOC2 (5 × 10^5^ cells) in 100 µL PBS were injected subcutaneously into the right flank. Tumor volume (mm^3^) was measured every 3 days and calculated as follows: (length × width^2^)/2.

#### Bone marrow neutrophil isolation and adoptive transfer

Mouse bone marrow neutrophils were isolated by density gradient centrifugation as previously described (21). Clarified neutrophils (5 × 10^6^ cells) were administered intravenously to the indicated groups.

#### *In vivo* treatments

Seven days after tumor implantation, mice received intraperitoneal injections of saline, rmCCL3 (5 µg per mouse; BioLegend, cat. #593804), anti-mouse Ly6G (200 µg per mouse; clone 1A8; Selleck, cat. #A2158; RRID:AB_3677246), or anti-mouse PD-1 (200 µg per mouse; Bio X Cell, cat. #BE0146; RRID:AB_10949053). Treatments were administered every 3 days for a total of five doses.

#### Neutrophil depletion

Neutrophils were depleted using anti-mouse Ly6G monoclonal antibody (clone 1A8; Selleck, cat. #A2158; RRID:AB_3677246) administered intraperitoneally at 200 µg per mouse every 3 days for a total of five doses. Control mice received a matched isotype control antibody (rat IgG2a, clone 2A3; Selleck, cat. #A2123; RRID:AB_3644245) using the same dose, route, and schedule.

#### Endpoints and tissue collection

One day after the fifth injection, mice were euthanized, and tumors were harvested for weight measurement and downstream analyses. All tumor volumes remained below the 2,000 mm^3^ endpoint specified in the animal permit.

#### Flow cytometry

Harvested tumors were dissociated into single-cell suspensions and stained with fluorochrome-conjugated monoclonal antibodies against CD45 (1:100; Thermo Fisher Scientific, cat. #12-0451-82; RRID:AB_465668), CD3 (1:100; Thermo Fisher Scientific, cat. #11-0032-82; RRID:AB_2572431), and CD8a (1:100; Thermo Fisher Scientific, cat. #17-0081-82; RRID:AB_469335). For neutrophil depletion validation, tumor digests were additionally stained for CD11b (1:100; Thermo Fisher Scientific, cat. #11-0112-41; RRID:AB_11042156) and Ly6G (1:100; Thermo Fisher Scientific, cat. #47-9668-82; RRID:AB_2802291) to quantify CD11b^+^ Ly6G^+^ neutrophils. Data were acquired on a BD C6 flow cytometer.

A consolidated key resources table, including antibodies, reagents, cell lines, animals, software, and algorithms, is provided in Supplementary Table S2.

### Statistical analysis

All statistical analyses and visualizations were performed in R (version 4.3.1). Comparisons of numerical variables between groups (e.g., response status, cell clusters) were performed using the two-sided Wilcoxon rank-sum test or *t* test, as appropriate for the data distribution. DEGs between cell groups were identified using a two-sided Wilcoxon rank-sum test, with *P* values adjusted for multiple comparisons using the Benjamini–Hochberg method. Associations between two categorical variables were assessed using Fisher’s exact test. Correlations between cell subpopulation fractions and radiologic response rates were evaluated using Spearman’s rank correlation. ROC curves were generated, and AUCs were calculated using the pROC package (version 1.18.5; RRID:SCR_024286) in R with default parameters. Statistical significance was set at a *P* value or adjusted *P* value < 0.05.

## Results

### Patient characteristics and treatment efficacy

**Figure 1.**
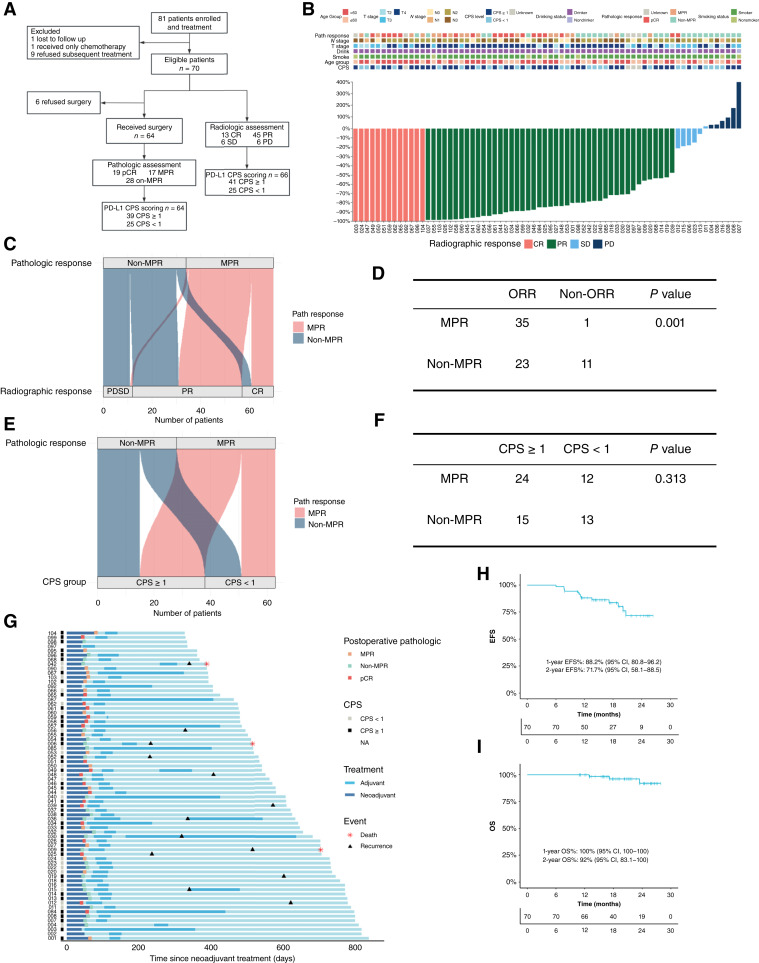
Study design and clinical efficacy of neoadjuvant toripalimab plus chemotherapy in HPSCC. **A,** CONSORT flow diagram illustrating patient recruitment, treatment allocation, and analysis populations. A total of 70 eligible patients were included in the intention-to-treat analysis, and 64 patients underwent surgery. **B,** Waterfall plot showing the best percentage change in maximum tumor diameter from baseline for each patient (*n* = 70). Colors indicate radiographic response according to Response Evaluation Criteria in Solid Tumors, version 1.1. **C,** Sankey diagram illustrating the concordance between radiographic response categories [CR, partial response (PR), stable disease/PD] and pathologic response categories (MPR vs. non-MPR) in the surgical cohort (*n* = 64). **D,** Contingency table analysis shows that patients who achieved an objective radiographic response (defined as CR or PR) were significantly more likely to reach MPR (two-sided Fisher’s exact test; *n* = 64 surgically treated patients). **E,** Sankey diagram showing the relationship between pretreatment PD-L1 CPS status (≥1 vs. <1) and pathologic response (*n* = 64). **F,** Comparison of MPR rates stratified by PD-L1 CPS status. The *P* value was calculated using a two-sided Fisher’s exact test (*n* = 64). **G,** Swimmer plot depicting the clinical course for each patient (*n* = 70), including the duration of neoadjuvant therapy, timing of surgery, and follow-up status (recurrence or death). **H,** Kaplan–Meier OS curve for all treated patients (*n* = 70). **I,** Kaplan–Meier EFS curve for all treated patients.

Between March 2023 and July 2024, 81 patients were enrolled; 70 met eligibility and completed two cycles of neoadjuvant toripalimab plus paclitaxel and nedaplatin. All 70 patients were included in the primary efficacy and safety analyses. Subsequently, 64 underwent surgery and had specimens available for pCR/MPR assessment (Fig. 1A). Neoadjuvant therapy yielded an objective response rate of 82.7%. This included a complete response (CR) rate of 18.6% and a partial response rate of 64.2%, whereas 8.6% of patients had stable disease and 8.6% experienced progressive disease (PD). Overall, 91.5% proceeded to surgery; among the 64 resected patients, 29.7% achieved pCR [19/64; 95% confidence interval (CI), 18.9%–42.7%]. The MPR rate was 56.3% (36/64; 95% CI, 43.3%–68.4%). PD-L1 CPS was evaluable in 66/70 (92.9%), with 62.1% of these patients having a CPS ≥1 (Fig. 1B). In the 64 surgical cases, we assessed concordance between pathologic response and radiographic response, PD-L1 CPS, T stage, and N stage. Most patients who achieved a radiographic CR also attained an MPR, whereas only one patient with radiographic PD achieved an MPR (Fig. 1C). Patients who achieved an objective radiographic response were significantly more likely to attain MPR (*P* = 0.001, Fig. 1D). MPR was numerically higher with CPS ≥1, but not significant (*P* = 0.313, Fig. 1E and F). Furthermore, neither T stage nor N stage showed a significant association with pathologic response (Supplementary Fig. S1A–S1D).

The swimmer plot illustrates the treatment and follow-up trajectory for the entire cohort. As of June 16, 2025, 13 patients had experienced recurrence (3 local, 1 distant, 9 nodal), and 3 patients had died, all due to disease progression (Fig. 1G). The median age of the cohort was 63 years (range, 42–75), and all patients were male. A high proportion of patients had a history of smoking (88.5%) or alcohol consumption (95.7%). At baseline, 62.9% of patients presented with T4 stage tumors, and the vast majority (82.9%) had lymph node metastasis. The primary tumor was most commonly located in the pyriform sinus (74.3%; see Supplementary Table S3 for details). AEs during neoadjuvant treatment were predominantly grades 1 to 2 and manageable. Only 4.3% of patients experienced grade 3 immune-related pneumonitis, which led to surgical delay (Supplementary Table S4). Overall, the treatment regimen was safe and well tolerated. As of June 16, 2025, the median follow-up duration was 19.1 months (range, 10.9–28 months). The 1- and 2-year OS rates were 100% (95% CI, 100–100) and 93% (95% CI, 83.1–100), respectively. The 1- and 2-year EFS rates were 88.2% (95% CI, 80.8–96.2) and 71.1% (95% CI, 58.1–88.5), respectively (Fig. 1H and I). Prognostic analysis stratified by CPS status revealed no significant differences in either OS (*P* = 0.75, Supplementary Fig. S1E) or EFS (*P* = 0.79, Supplementary Fig. S1F) between the CPS ≥1 and CPS <1 groups.

### Single-cell profiling reveals baseline TME features associated with nCIT response

**Figure 2.**
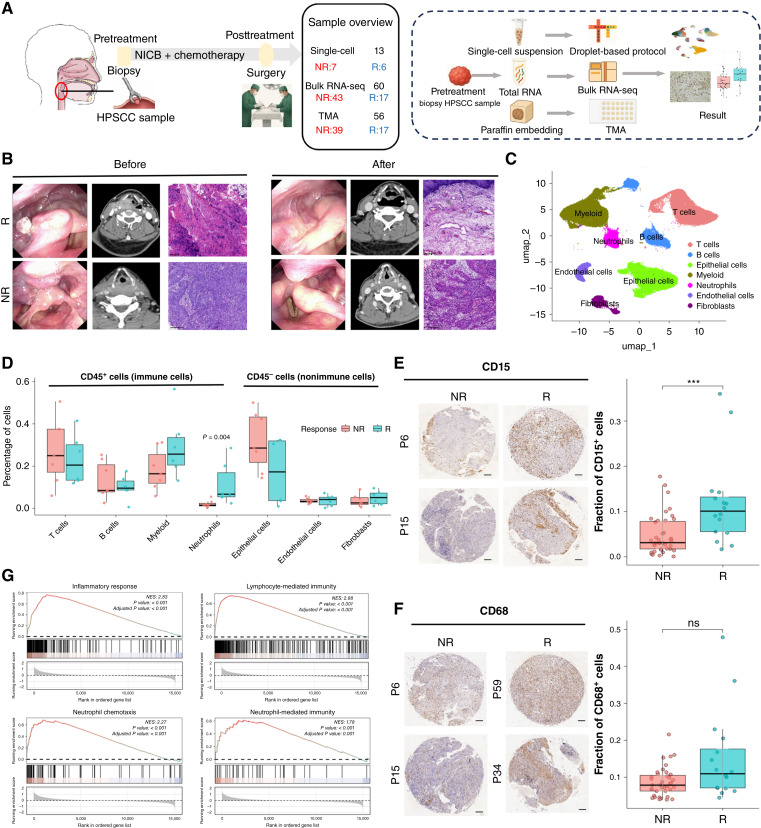
Single-cell transcriptomic landscape of the HPSCC TME before nCIT. **A,** Schematic diagram of the study design. **B,** Pre- and posttreatment CT scans and H&E-stained tissue sections from one R and one NR. Scale bar, 400 μm. **C,** UMAP visualization of 97,099 high-quality cells from 13 pretreatment HPSCC tumors, colored by major cell lineage. **D,** Box plot comparing the relative abundances of major cell lineages in the TME between Rs (*n* = 6) and NRs (*n* = 7). The middle line indicates the median, and the box denotes the interquartile range (IQR). **E,** Neutrophil infiltration in the IHC validation TMA cohort (*n* = 56), with a box plot quantifying the percentage of positive cells. **F,** Macrophage infiltration in the IHC validation TMA cohort (*n* = 56), with a box plot quantifying the percentage of positive cells. Two-sided Wilcoxon rank-sum test, ***P < 0.001; ns, no significant difference. Scale bar, 200 μm. **G,** GSEA of hallmark pathways.

To elucidate drivers of nCIT efficacy, we prospectively collected pretreatment primary tumor biopsies. Pretreatment tumor samples from 13 patients were subjected to scRNA-seq, whereas biopsies from 60 patients were used for bulk RNA-seq, and samples from 56 patients were used for TMA analysis with IF and IHC (Fig. 2A). Based on postsurgical pathology, the scRNA-seq cohort was classified as Rs (*n* = 6) or nonresponders (NR, *n* = 7). Rs achieved pCR (no residual tumor in the hypopharynx); NRs had residual tumor after surgery. After nCIT, R lesions shifted from tumor-like to near-normal mucosa (Fig. 2B). Radiologic responses and follow-up status of the scRNA-seq subcohort (*n* = 13) are summarized in Supplementary Fig. S2A. Pathologic response aligned with greater CT tumor shrinkage (Supplementary Fig. S2B).

After quality control, we obtained 97,099 single-cell transcriptomes from 13 pretreatment tumor samples. Using canonical markers, cells were annotated as T, B, myeloid, neutrophils, stromal (fibroblasts/endothelial), or epithelial (Fig. 2C; Supplementary Fig. S2A and S2B). We compared major cell type fractions between R and NR pretreatment samples (Fig. 2D; Supplementary Fig. S2C and S2D). Notably, the apparent increase in the total T-cell proportion in the NR group in Fig. 2D did not reach statistical significance at the per-patient level. We therefore next examined T-cell phenotypes and functional states in dedicated downstream analyses. Myeloid cells and neutrophils were more abundant in R pretreatment tumors than in NR. Differential expression analysis of the scRNA-seq data revealed that inflammatory/chemotaxis-related genes were significantly upregulated in the R group compared with the NR group, suggesting enhanced myeloid activation and neutrophil recruitment (Supplementary Fig. S2F and S2H). We validated this by IHC in pretreatment specimens. The IHC results confirmed that neutrophil (CD15^+^) infiltration was significantly higher in the R group samples than in the NR group, whereas macrophage (CD68^+^) infiltration showed no significant difference between the two groups (Fig. 2E and F).

We performed bulk RNA-seq DEG analysis on pretreatment samples comparing pCR versus non-pCR (Supplementary Fig. S2G). In the R group, upregulated genes were mainly related to inflammation/chemotaxis and immune activation, whereas the upregulated genes in the NR group were associated with epithelial differentiation/cell junction and metabolic detoxification. GSEA of DEGs showed immune pathways enriched in R, including inflammatory response, lymphocyte programs, neutrophil chemotaxis, and neutrophil-mediated immunity (Fig. 2G). Bulk data agreed with scRNA-seq, supporting a more proinflammatory TME in R that facilitates effector cell infiltration.

### Epithelial, B cell, and macrophage programs promote neutrophil recruitment

**Figure 3.**
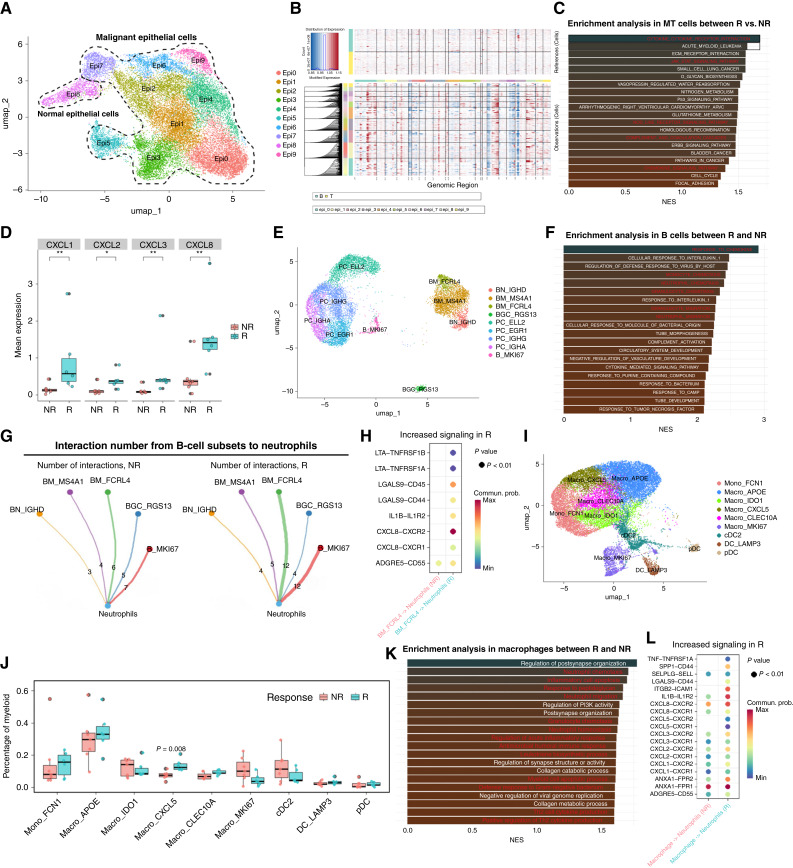
Malignant epithelial cells, B cells, and macrophages in Rs promote neutrophil recruitment. **A,** UMAP visualization of all epithelial cells, colored by cluster identity. Cells within the dashed line were identified as MT based on copy-number variation analysis. **B,** Single-cell CNV heatmap, with each row representing one epithelial cell and each column representing a chromosomal region. **C,** Pathways enriched among DEGs in MT cells from R vs. NR groups (GSEA). **D,** Box plot quantifying the expression levels of key chemokines (R, *n* = 6; NR, *n* = 7). *, *P* < 0.05; **, *P* < 0.01. **E,** UMAP of B cells, colored by subtype identity. **F,** Pathways enriched among DEGs in B cells from R vs. NR groups (GSEA). **G,** Number of key ligand–receptor pairs from B-cell subsets to neutrophils in different response groups. **H,** CellChat bubble heatmap showing predicted interactions between BM_FCRL4 B cells (ligand) and neutrophils (receptor) in R vs. NR groups. **I,** UMAP of myeloid cells, colored by subset identity. **J,** Box plot comparing the proportions of each myeloid cell subset between R and NR patients. **K,** Bar plot of GSEA results for pathways enriched in DEGs between total macrophages from R and NR patients. **L,** CellChat bubble heatmap showing predicted interactions between macrophages (ligand) and neutrophils (receptor) in R vs. NR groups.

We explored drivers of neutrophil enrichment. Lineage-specific differential expression analysis showed higher chemotactic/inflammatory programs in R, most prominently in epithelial cells, B cells, and macrophages, implicating them as key contributors to neutrophil recruitment signals (Supplementary Fig. S2H). We analyzed these lineages in parallel: we reclustered epithelial cells into 10 subsets (Epi0–Epi9) and used inferCNV/CytoTRACE to label Epi8 as normal epithelium and the remainder as malignant (MT) cells (Fig. 3A and B; Supplementary Fig. S3A and S3B). Enrichment implicated immune-interaction pathways, including cytokine–receptor interaction, chemokine signaling, complement, NOD-like receptor signaling, and JAK–STAT (Fig. 3C). Specifically, multiple chemokines were upregulated in the MT cells of R group patients, such as *CXCL1*/*CXCL2*/*CXCL3*/*CXCL8* (Fig. 3D; Supplementary Fig. S3C). CellChat predicted stronger MT-to-neutrophil chemotactic signaling in R versus NR (Supplementary Fig. S3D). CXCL1/2/3/8 is known to promote the recruitment of immune cells such as neutrophils and macrophages, particularly mobilizing neutrophils to infiltrate tumors (22). *CXCL1* and *CXCL8* can also promote angiogenesis, potentially improving the delivery of chemotherapy and immunotherapy to tumors (23). Together, these data suggest that tumor cells in Rs may help establish a proinflammatory TME that supports immune infiltration and function.

We reclustered B cells into nine subsets (Fig. 3E), including naïve, memory, germinal-center, plasma, and proliferative B cells. To assess the role of B cells in treatment outcomes, we performed GSEA on B cells from the R and NR groups. The results showed that B cells from R patients were enriched for GO biological processes related to neutrophil chemotaxis, migration, and immune response (Fig. 3F). Enrichment of neutrophil–chemotaxis pathways in Rs indicates a more inflammatory, immunotherapy-permissive TME. Further cell communication analysis showed that in R patients, memory B cells—particularly the BM_FCRL4 subset—had more interactions with neutrophils (Fig. 3G and H). Studies have shown that in NSCLC, an FCRL4^+^FCRL5^+^ B-cell subset (termed “atypical memory B cells”) is also modestly enriched in patients who achieve MPR (24). *FCRL4* and *FCRL5* encode the Fc receptors for IgA and IgG, respectively, and are drivers of human memory B-cell activation. Moreover, compared with the NR group, BM_FCRL4 cells in R patients interacted with other immune cells in the TME through multiple signaling pathways. Specifically, pathways such as *LTA*, IL-16, *ICAM1*, MHC I/II, *CXCL10*, *CD86*, and *CCL5* play active roles in T-cell activation, effector immune cell recruitment, and enhanced antigen presentation (Supplementary Fig. S3F and S3G). In our bulk RNA-seq data, the GSEA signature scores calculated for BM_FCRL4 cells and neutrophils showed a significant positive correlation between these two cell populations (Supplementary Fig. S3E). In addition, recent studies have emphasized the role of B cells and tertiary lymphoid structures (TLS) in checkpoint blockade efficacy and T cell–mediated antitumor immunity (25). We found that TLS signature scores for each B cell subset were significantly higher in R patients than in NR patients, with the difference especially pronounced in the two memory B-cell subsets (BM_MS4A1 and BM_FCRL4; Supplementary Fig. S3H).

Using classical markers, we subdivided the myeloid cells into nine subsets: one monocyte subset, five macrophage subsets, and three dendritic cell subsets (Fig. 3I). One specific macrophage subset (Macro_CXCL5) was significantly enriched in tumors of R group patients (Fig. 3J). Compared with other monocyte–macrophage subsets, Macro_CXCL5 cells express *CXCL1*/*CXCL3*/*CXCL5*/*CXCL8*, *IL6*, and other proinflammatory chemotactic genes, defining this subset as a proinflammatory/chemotactic type of tumor-associated macrophage (Supplementary Fig. S3I). We performed GSEA on the differential genes between R and NR groups for the macrophage population (Fig. 3K). The R group was enriched for acute proinflammatory pathways such as “neutrophil chemotaxis/migration/activation” and “regulation of acute inflammatory response.” Further cell communication analysis revealed that R group macrophages had a stronger potential chemotactic effect on neutrophils (Fig. 3L). This suggests that R macrophages promote neutrophil recruitment and acute inflammation, supporting effector cell infiltration.

### Characteristics of the Neu_CCL3 neutrophil subset and its predictive value for immunotherapy response

**Figure 4.**
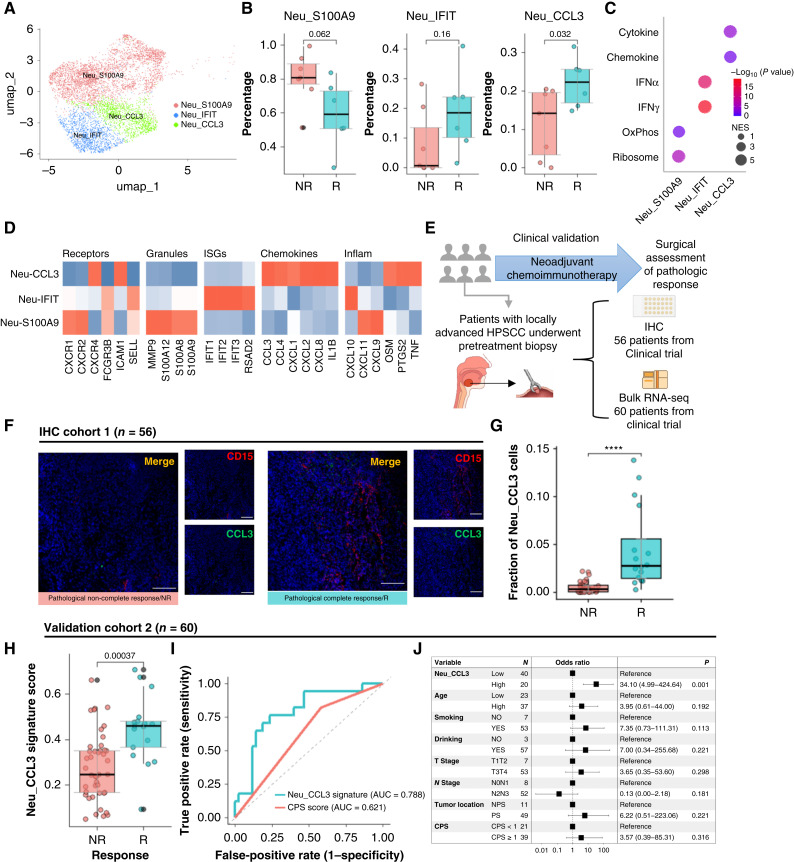
The Neu_CCL3 subset is enriched in Rs, and its signature score robustly predicts immunotherapy outcomes. **A,** UMAP of neutrophils, colored by subset identity. **B,** Box plot comparing the proportions of each neutrophil subset between R and NR patients. **C,** Bubble chart showing pathways enriched among DEGs for each neutrophil subset (GSEA; only significantly enriched pathways are shown). **D,** Heatmap showing the normalized expression of canonical marker genes for each neutrophil subset. **E,** Schematic diagram of the HPSCC validation cohort receiving nCIT. **F** and **G,** Representative dual-marker multiplex IF (mIF) images of pretreatment tumors from R and NR patients in mIF cohort 1 (*n* = 56; scale bar, 200 μm; **F**) and the corresponding quantification (**G**). Each point in the box plot represents one sample. ****, *P* < 0.0001. **H,** Neu_CCL3 signature scores in validation cohort 2 (*n* = 60), compared between R and NR patients. **I,** Area under the ROC curve (AUC) for pCR prediction by the Neu_CCL3 signature and PD-L1 CPS score in validation cohort 2. **J,** Forest plot of a multivariate logistic regression analysis in validation cohort 2 showing the association of clinical factors and the Neu_CCL3 signature with pCR.

Collectively, these data indicate that multiple R-associated programs—including MT epithelial chemokines, TLS-active memory B-cell states, and proinflammatory macrophage signaling—converge on enhanced neutrophil recruitment/activation in the pretreatment TME. Because neutrophils were enriched in Rs (Fig. 2D), we reclustered tumor neutrophils and identified three subsets. Neu_CCL3 cells were enriched in Rs and scarce in NRs (Fig. 4A and B). Neu_CCL3 cells overexpressed multiple chemokines, such as *CXCL8*, *CCL3*, and *CCL4* (Fig. 4D). Enrichment analysis of Neu_CCL3 DEGs confirmed cytokine/chemokine pathways (Fig. 4C). To test clinical predictiveness, we analyzed pretreatment tumors by IHC (*n* = 56) and bulk RNA-seq (*n* = 60; Fig. 4E). IF for CCL3 and CD15 in 56 pretreatment tumors showed higher CCL3^+^CD15^+^ density in pCR cases (Fig. 4F and G). Bulk RNA-seq confirmed higher Neu_CCL3 signature scores in Rs (Fig. 4H and I). The Neu_CCL3 signature outperformed PD-L1 CPS for pCR prediction (AUC 0.788 vs. 0.621, Fig. 4I). In multivariable logistic regression, each 1-SD increase in Neu_CCL3 score was associated with higher odds of pCR (OR = 34.10, 95% CI, 4.99–424.61, *P* = 0.001, Fig. 4J). In contrast, other clinical factors such as age, smoking, alcohol use, T stage, N stage, tumor site, and CPS were not statistically significant in the multivariable model (*P* > 0.05). Thus, the Neu_CCL3 signature independently predicted response and outperformed standard clinical variables.

We validated the Neu_CCL3 signature in three published anti–PD-1 plus chemotherapy cohorts with pretreatment transcriptomes (Supplementary Fig. S4A). In the NSCLC cohort (*n* = 24), Rs had significantly higher scores (*P* = 0.018) with good discrimination (AUC = 0.793, Supplementary Fig. S4B and S4C). In two melanoma cohorts (*n* = 49 and *n* = 41), Rs similarly showed higher scores (*P* = 0.030 and *P* = 0.021, respectively) and consistent discriminative performance (AUC = 0.723 and 0.711, Supplementary Fig. S4D–S4G). Overall, the Neu_CCL3 signature predicted response across cancer types, consistent with our internal validation.

### Neu_CCL3 cells enhance the efficacy of anti–PD-1 therapy *in vivo*

**Figure 5.**
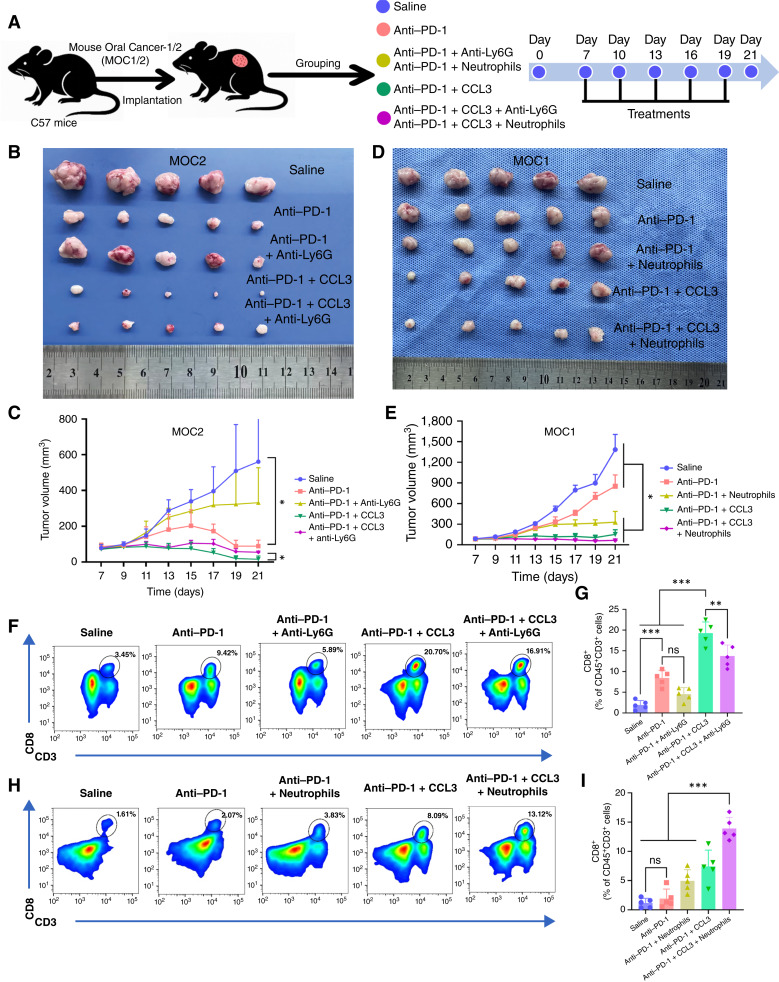
Neu_CCL3 enhances responsiveness to anti–PD-1 therapy in syngeneic MOC1 and MOC2 tumor models. **A,** Schematic of the experimental design and treatment schedule. Neutrophil depletion was performed using anti-Ly6G (clone 1A8; 200 μg i.p. every 3 days for five doses) and was validated by flow cytometry. **B** and **C,** MOC2 model: (**B**) representative images of excised subcutaneous tumors at endpoint; **C,** tumor growth curves (*n* = 5 mice per group), shown as mean ± standard deviation (SD). **D** and **E,** MOC1 model: (**D**) representative images of excised subcutaneous tumors at endpoint; **E,** tumor growth curves (*n* = 5 mice per group), shown as mean ± SD. **F** and **G,** MOC2 model: (**F**) representative flow cytometry plots of intratumoral CD8^+^ T cells (gated on live CD45^+^CD3^+^ cells) across the indicated treatment groups and (**G**) quantification of intratumoral CD8^+^ T-cell frequencies. **H** and **I,** MOC1 model: (**H**) representative flow cytometry plots of intratumoral CD8^+^ T cells (gated on live CD45^+^CD3^+^ cells) across the indicated treatment groups and (**I**) quantification of intratumoral CD8^+^ T-cell frequencies. Statistical analyses were performed using a two-sided Student *t* test. ns, not significant; *, *P* < 0.05; **, *P* < 0.01; ***, *P* < 0.001. Flow cytometry plots are representative of one mouse per group; quantification includes all mice.

To test whether Neu_CCL3 augments anti–PD-1 efficacy, we performed neutrophil depletion and CCL3 supplementation in MOC1 and MOC2 models (Fig. 5A). MOC1 is highly immunogenic, whereas MOC2 is more immunosuppressive (26–28). Anti–PD-1 alone modestly inhibited tumor growth in both models. The effect was stronger in MOC1 than in MOC2. A pilot depletion experiment confirmed that anti-Ly6G treatment markedly reduced intratumoral CD11b^+^Ly6G^+^ neutrophils (Supplementary Fig. S5A), supporting effective neutrophil depletion in our *in vivo* setting. Neutrophil depletion partially abrogated the efficacy of the PD-1 antibody, but with exogenous CCL3 supplementation, tumor volume and weight both declined significantly, reversing the tumor growth caused by neutrophil depletion and suggesting that CCL3 can enhance the efficacy of PD-1 immunotherapy (Fig. 5B–E; Supplementary Fig. S5B and S5C). In MOC1, neutrophil depletion reduced anti-PD-1–induced CD8^+^ T-cell infiltration; CCL3 supplementation reversed this effect (Fig. 5F and G). Similarly, in the MOC2 model, after administering neutrophils, combined PD-1 and CCL3 treatment likewise significantly promoted CD8^+^ T-cell infiltration and enhanced the antitumor effect (Fig. 5H and I). In summary, CCL3^+^ neutrophils demonstrated the potential to enhance PD-1 therapy efficacy in both immune microenvironment contexts, possibly exerting a synergistic antitumor effect by enhancing T-cell infiltration and activation.

### Preexisting CD69-high CD8 T cells with TRM-like features are associated with treatment efficacy

**Figure 6.**
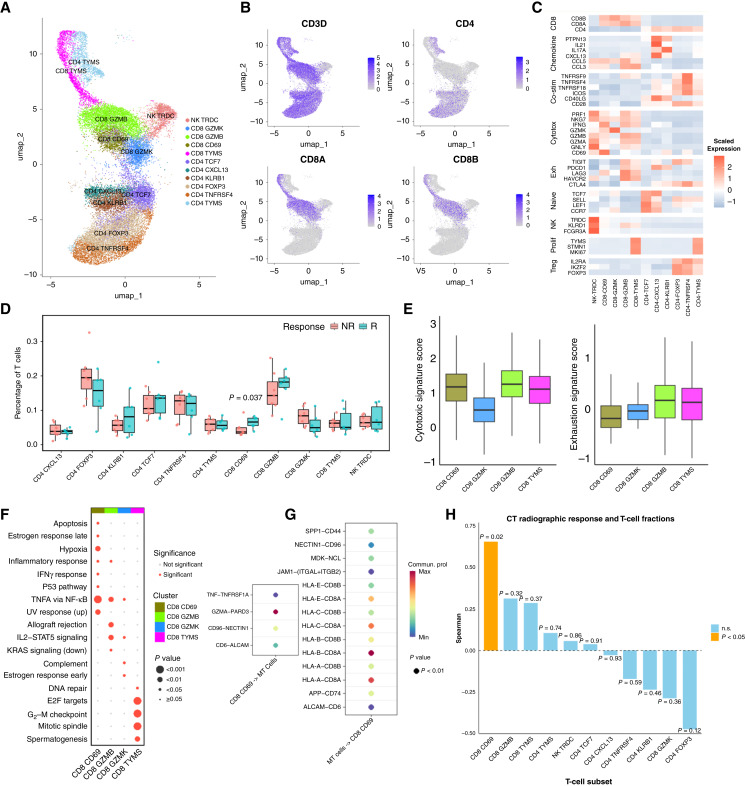
An early-activated CD8_CD69 T cell subset is associated with response to nCIT. **A,** UMAP of T and NK cells, colored by subset identity. **B,** UMAP showing the expression of canonical marker genes in the major T-cell phenotypes. **C,** Heatmap showing the expression of canonical marker genes (normalized) across each T/NK cell subset. **D,** Box plot comparing the proportions of each T/NK cell subset (as a percentage of total T/NK cells) between R and NR patients (R, *n* = 6; NR, *n* = 7; each dot represents one patient). **E,** Box plots showing the cytotoxicity (left) and exhaustion (right) gene signature scores for each CD8^+^ T-cell subset (signature genes are detailed in “Patients and Methods”). **F,** Bubble chart showing enriched pathways among DEGs for each CD8^+^ T-cell subset (GSEA; only significantly enriched pathways are shown). **G,** CellChat bubble heatmap showing significant ligand–receptor interactions between MT cells and CD8_CD69 T cells. **H,** Bar graph showing the Spearman correlation coefficients between the infiltration fraction of each T/NK cell subset and the percentage of tumor shrinkage after neoadjuvant therapy.

As antitumor immunity elicited by PD-1 blockade is largely mediated by T cells, to identify the key T-cell subset mediating the nCIT response, we reclustered the T/NK cells into 11 subsets (Fig. 6A–C). CD8_CD69 T cells were more abundant in pretreatment tumors from Rs (Fig. 6D). At the whole T-cell level, Rs showed a trend toward higher cytotoxic program activity with broadly comparable exhaustion (Supplementary Fig. S6A). In the bulk RNA-seq validation cohort, ssGSEA increased lymphocyte/cytotoxic and T cell–inflamed activity in Rs (Supplementary Fig. S6C), and cytotoxic activity was positively associated with the Neu_CCL3 score (Supplementary Fig. S6B). CD8_CD69 T cells showed higher cytotoxic program activity and relatively lower exhaustion-associated signatures based on module scoring (Fig. 6E). Taken together, CD8_CD69 T cells highly expressed the early activation and tissue-retention marker CD69 and exhibited a cytotoxic, less-exhausted transcriptional profile, consistent with a CD69-high tissue-retention memory (TRM)-like CD8 T-cell state rather than definitively CD103-defined TRM (29). TRM-like CD8 T-cell populations have been associated with local immune surveillance and favorable outcomes to immune checkpoint blockade in multiple tumor types (30). DEG enrichment showed that TNF, p53, and apoptosis pathways were upregulated in CD8_CD69 T cells (Fig. 6F). CD8_CD69 T cells engaged tumor-killing pathways (*TNF*-*TNFRSF1A*, *GZMA*-*PARD3*, *CD6*-*ALCAM*; Fig. 6G). Tumor cells presented antigen via class I HLA, supporting CD8 T-cell activity (Fig. 6G). Importantly, among all T cell subsets, only the infiltration level of CD8_CD69 T cells showed a significant positive correlation with the percentage of tumor shrinkage after nCIT (R = 0.653, *P* < 0.05), whereas no such correlation was observed for the other subsets (Fig. 6H). These results indicate that CD8_CD69 T cells are early-activated CD8^+^ T cells and represent a promising biomarker for predicting immunotherapy response.

### Neu_CCL3–CD8–macrophage cross-talk supports antitumor immunity

**Figure 7.**
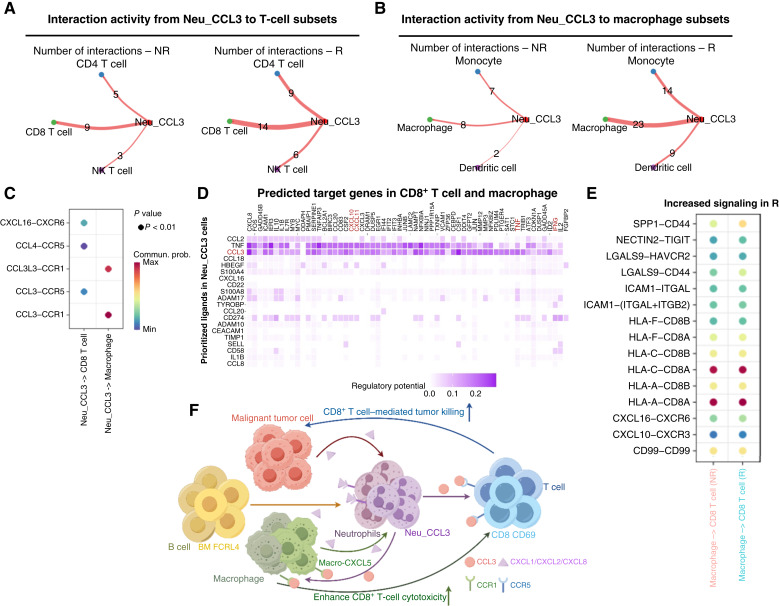
Interactions between the Neu_CCL3 subset and T-cell or macrophage subsets. **A,** Number of significant ligand–receptor pairs from Neu_CCL3 cells to T-cell subsets in different response groups. **B,** Number of significant ligand–receptor pairs from Neu_CCL3 cells to macrophage subsets in different response groups. **C,** Bubble heatmap showing ligand–receptor interactions between Neu_CCL3 cells and CD8^+^ T cells and macrophages. **D,** Heatmap showing the activity (left) and regulatory potential (right) of top ligands in Neu_CCL3 cells that drive the antitumor potential of CD8^+^ T cells and macrophages. **E,** Bubble heatmap showing ligand–receptor interactions between macrophages and CD8^+^ T cells. **F,** Summary diagram of the pre-nCIT TME dynamics in patients with HPSCC who achieved pCR, by Figdraw.

We then examined Neu_CCL3-mediated cross-talk with CD8^+^ T cells and macrophages. Using CellChat to perform interaction network analysis with Neu_CCL3 as the sender, we found that interactions between Neu_CCL3 and immune effector subsets (CD8^+^ T cells, macrophages) were greater in number and strength than with other cell types and were more pronounced in the R group (Fig. 7A and B). Neu_CCL3 preferentially signaled via *CCL3*/*CCL3L3*/*CCL4*–*CCR1*/*CCR5* and *CXCL16–CXCR6* (Fig. 7C), consistent with immune recruitment/retention. Ligand–target gene inference further showed that these signals preferentially drove the expression of immune activation/effector genes such as *CXCL9*, *CXCL10*, *TNF*, and *IFNG* in the receptor cells (Fig. 7D). In Rs, macrophage → CD8^+^ T interactions upregulated *ICAM1–LFA1*,* HLA-A*/*C–CD8*, *CXCL10–CXCR3*, and *CXCL16–CXCR6* (Fig. 7E). Notably, inhibitory/regulatory axes such as *NECTIN2–TIGIT*, *LGALS9–TIM-3*, and *SPP1–CD44* were also concomitantly enhanced, suggesting a compensatory brake in the context of strong activation. In summary, the data support a model in which MT epithelial cells, B cells, and macrophages upregulate chemotactic/inflammatory molecules to recruit and sustain neutrophils. Neu_CCL3 cells, in turn, attract CD8^+^ T cells and proinflammatory macrophages into the tumor via chemokine signals. Subsequently, adhesion and antigen-presentation axes stabilize the immunologic synapse and target recognition, leading to enhanced CD8^+^ T-cell cytotoxicity and tumor cell clearance (Fig. 7F). This positive feedforward loop of “chemotaxis–adhesion/presentation–effector” is consistent with the “hot” TME characteristics observed in Rs.

## Discussion

In this prospective, single-arm phase II trial, toripalimab plus albumin-bound paclitaxel and nedaplatin showed clinically meaningful activity with a manageable safety profile in patients with locally advanced HPSCC. The trial met its primary endpoint, with a pCR of 29.7% and an MPR of 56.3% among 64 evaluable patients, comparable with prior neoadjuvant studies in HNSCC. For example, multiple clinical studies have shown that neoadjuvant anti–PD-1/PD-L1 monotherapy can achieve MPR in roughly 20% to 30% of patients (31, 32). Moreover, earlier clinical trials indicated that nCIT in advanced HNSCC yields a pCR rate of 15% to 40% and an MPR rate of 30% to 60% (4, 8, 33–38). One phase II trial in head and neck squamous carcinoma reported an overall MPR rate of 63%; however, no such responses were observed in the HPSCC subset (4). The efficacy observed in our study was also notably better than the approximately 13% to 27% pCR rate achieved by conventional chemotherapy alone in locally advanced HNSCC (39–41). Meanwhile, based on cross-trial evidence, a meta-analysis found that the incidence of grade 3 to 4 AEs with neoadjuvant immunotherapy was around 8.4%, markedly lower than the 37% with chemotherapy alone (42); however, such indirect comparisons should be interpreted cautiously given differences in patient populations, regimens, and reporting standards, and because toxicity may vary by sex. In our study, the incidence of ≥ grade 3 treatment-related AEs was 4.3%, well below previously reported rates, supporting a manageable safety profile of this regimen in our all-male cohort (43). However, because no female patients were enrolled and sex-related differences in treatment toxicity and immune-related AEs have been reported—with several analyses suggesting a higher risk of severe AEs in women—these safety findings should be interpreted with caution and require confirmation in cohorts including women with prespecified sex-stratified safety analyses (44, 45). Moreover, perioperative nCIT has been shown to improve EFS (46). Overall, these results support integrating immunotherapy into multimodality treatment for locally advanced HPSCC.

Our study focuses on HPSCC, an understudied subtype with a poor prognosis. Although often grouped within HNSCC, HPSCC’s anatomy and aggressiveness warrant separate treatment considerations (47). To our knowledge, this is the first phase II trial of nCIT in HPSCC with immune microenvironment profiling. This work fills a gap in the existing research and holds significant clinical implications for guiding neoadjuvant therapy in hypopharyngeal carcinoma. However, PD-L1 CPS did not reliably distinguish benefit in our cohort, underscoring the need for alternative biomarkers (48, 49). Given PD-L1’s known limitations (dynamic expression, heterogeneity) and only moderate predictive value (50–52), we sought alternative determinants of response.

The results of the single-cell transcriptomic analysis have enriched our understanding of the tumor immune microenvironment. Baseline TME state largely determines nCIT efficacy (53, 54). Tumor epithelial cells are not only sources of antigens and inflammatory signals but also influence the recruitment and polarization of innate immune cells by secreting various cytokines (55). The bidirectional “conspiratorial” relationship between tumor cells and infiltrating myeloid cells (e.g., neutrophils and macrophages) can shape either a protumor or an anti-TME (56). We found that in pretreatment pCR tumors, MT epithelial cells, B cells, and macrophages produced neutrophil-attracting chemokines. These tumors seemed to establish a proinflammatory, immune-permissive TME that supports immune infiltration. Our study also highlights the role of B cells and tumor-associated TLSs in antitumor immunity. A large body of literature indicates that the presence of intratumoral B cells and TLSs is significantly associated with improved patient prognosis and immunotherapy response (57, 58). We found that the TLS score for a memory B-cell subset (BM_FCRL4) was significantly higher in pCR patients. FCRL4^+^ B cells are associated with chronic inflammation and tissue residency (59). These cells may further establish a positive feedback loop: By expressing chemokines such as *CXCL8*, they promote and sustain neutrophil infiltration. Notably, our study cohort had a unique etiologic background: more than 80% of the patients had a history of smoking and alcohol use. Prolonged physicochemical irritation is a major risk factor for HPSCC and can induce chronic inflammation (60). This chronic inflammatory background could result in two fundamentally different immune outcomes: in some patients, the immune system successfully maintains surveillance and attack against the tumor, forming the “immune-permissive” TME we observed that is rich in B cells, TLS, and proinflammatory signals, whereas in other patients, prolonged antigen exposure and inflammatory stimulation may lead to immune exhaustion or tolerance, resulting in an immunosuppressive “cold” TME (55). Therefore, patients’ exposure history is likely the fundamental reason why their TME exhibits a “preset” responsive state before treatment.

In parallel, we observed an R-enriched proinflammatory macrophage program (Macro_CXCL5) characterized by high expression of CXCL1/3/5/8 and IL6, together with stronger predicted chemotactic signaling toward neutrophils. These findings suggest that functionally specialized macrophage states—rather than total CD68^+^ density—may contribute to shaping an “immune-permissive” chemokine milieu. Beyond serving as upstream cues for neutrophil recruitment, BM_FCRL4-associated TLS activity and Macro_CXCL5 inflammatory programs may represent complementary biomarkers and potential intervention points, which warrant dedicated functional perturbation and combined predictive modeling in future studies.

Because R-associated chemotactic programs converged on neutrophil recruitment and the neutrophil–CCL3 axis is experimentally tractable, we dissected neutrophil heterogeneity and identified a proinflammatory CCL3-high subset (Neu_CCL3) as a key hub determining the efficacy of nCIT. Neu_CCL3 was enriched in pCR tumors and marked by high *CCL3* expression. Mechanistically, Neu_CCL3 may recruit effector T cells and monocytes/macrophages via *CCL3–CCR5*/*CCR1*, supporting its association with efficacy (61, 62). CCL3 is a classic inflammatory chemokine known to attract CCR5^+^ CD8^+^ T cells and monocytes into tissues and promote their activation (63). CellChat supported Neu_CCL3-to-CD8^+^ T-cell/macrophage recruitment signals. On the other hand, compared with currently used predictors such as PD-L1 expression, the Neu_CCL3 subset may more directly reflect the dynamic status of the tumor immune microenvironment. Across several trials, PD-L1 expression was not associated with improved pathologic response, indicating limited predictive utility (64, 65). Multicomponent immune profiles incorporating neutrophils and T cells can improve response prediction (15, 16, 66). In our cohort, the Neu_CCL3 score predicted pCR (AUC = 0.788) and outperformed PD-L1 CPS (AUC = 0.621). In multivariable logistic regression, Neu_CCL3 remained independently associated with pCR. PD-L1 failed to serve as a reliable marker because it only gauges a static, potentially inhibitory pathway (48, 67); by contrast, the Neu_CCL3 score succeeds because it captures the dynamic, functional “engine” activity of the antitumor immune network, providing a more comprehensive and accurate indicator of the tumor’s immune competence. In future studies, integrating Neu_CCL3 with complementary immune-context features—such as PD-L1, tumor mutational burden, TLS/B-cell activity (including BM_FCRL4-related states), and proinflammatory macrophage programs (e.g., Macro_CXCL5)—may enable a more robust multiparameter model to better identify the HPSCC population most likely to benefit from nCIT.

Neu_CCL3 may orchestrate effector recruitment via its chemokine network. Recruited CD8^+^ T cells included a CD8_CD69 subset enriched in Rs, with high cytotoxicity and low exhaustion. CD69 is a well-recognized marker of early T-cell activation and tissue residency and is commonly used as part of a TRM-like annotation in transcriptomic analyses, whereas CD103/ITGAE expression can be context-dependent in tumors (30, 68). As sentinels within tissues, TRM cells can mount rapid and robust responses to *in situ* antigens; their high levels of infiltration in multiple tumors are strongly associated with favorable prognosis and the efficacy of immune checkpoint inhibitors (69–71). Rs seemed to harbor preexisting functional, nonexhausted T cells. The recruitment and/or maintenance of these potent TRM cells may be driven by the chemokine environment established by the Neu_CCL3 subset, particularly via the CCL3–CCR5 axis as CCR5 is a key receptor mediating T-cell homing and activation (72–74). In mice, exogenous CCL3 enhanced anti–PD-1 efficacy, whereas neutrophil depletion blunted the benefit, supporting a causal contribution of CCL3^+^ neutrophils. Thus, response aligned with functionally competent (cytotoxic, less exhausted) TIL states rather than total T-cell abundance (75).

This study has several limitations. First, without a chemotherapy-only or ICI-only control arm, we cannot disentangle regimen-specific effects or formally test treatment interaction; Neu_CCL3 may therefore be prognostic rather than strictly predictive. Second, Neu_CCL3 was derived in a single-center nCIT setting; its transportability across treatment backbones, disease spectra, institutions, and assay platforms—and the calibration of clinically actionable thresholds—remains to be determined. Third, all enrolled participants were male, limiting generalizability and precluding assessment of sex-related differences in biomarker performance and treatment-related toxicity; thus, safety/tolerability conclusions should be interpreted in this context. Fourth, although we validated the *in situ* presence of CCL3^+^CD15^+^ cells by multiplex IF, additional work is needed to streamline clinically scalable workflows (e.g., routine IHC-based assays), establish cross-platform concordance, and align pathology response assessment with globally harmonized frameworks (e.g., PTE vs. RVT-based scoring and different tumor regression grade systems; ref. 76). Fifth, mechanistic validation focused on neutrophils; other immune components enriched in Rs (e.g., B cell– and macrophage-related programs) were not interrogated with comparable depth, and their contributions warrant dedicated follow-up. Sixth, the scRNA-seq cohort was modest (*n* = 13), and survival follow-up remains immature with few OS events; the study was not powered to robustly link pathologic response or Neu_CCL3 to long-term EFS/OS, underscoring the need for larger multicenter studies with longer follow-up and controlled comparators (77). Looking ahead, our findings open up new directions for HPSCC treatment. The top priority is to prospectively validate the Neu_CCL3 score in a phase III clinical trial, with the aim of translating it into clinical practice for selecting the patients most likely to benefit. A more revolutionary approach would be to explore whether “nonresponsive” Neu_CCL3-deficient “cold” tumors can be converted into “responsive” “hot” tumors through pharmacologic means—for example, by using TLR or STING agonists to induce a proinflammatory neutrophil phenotype. This would transform our biomarker from a passive predictive tool into an active therapeutic target.

In summary, our study identifies a CCL3^+^ proinflammatory neutrophil program associated with pathologic response to neoadjuvant toripalimab plus chemotherapy in locally advanced HPSCC, and the regimen demonstrated encouraging activity with a manageable safety profile in this all-male cohort. Building on this key mechanistic discovery, we developed and validated the Neu_CCL3 gene signature score, effectively filling the void left by the shortcomings of the PD-L1 biomarker and paving the way for precision immunotherapy in HPSCC and beyond.

## Supplementary Material

Supplementary File S1 Supplementary: Clinical trial protocol

Supplementary Figure S1 Comparison of clinical outcomes and survival analyses across different T/N stages and PD-L1 CPS status.

Supplementary Figure S2 Clinical overview, major lineage annotation, and differential expression analyses of the scRNA-seq discovery cohort.

Supplementary Figure S3 Epithelial CNV/stemness features, epithelial–neutrophil communication, and immune-cell states associated with response.

Supplementary Figure S4 Validation of Neu_CCL3 in pan-cancer nCIT cohorts.

Supplementary Figure S5 Anti-Ly6G depletion and tumor weights

Supplementary Figure S6 Functional characterization of T-cell states and lymphocyte-activity signatures in responders versus non-responders.

Supplementary Table S1 Representativeness of Study Participants

Supplementary Table S2 Key Resources Table

Supplementary Table S3 Patient characteristics

Supplementary Table S4 Treatment-related adverse events by grade (N=70)

## Data Availability

Raw scRNA-seq and bulk RNA-seq data generated in this study have been deposited in the Genome Sequence Archive for Human under accession HRA015910 and are publicly available. Publicly available datasets used for external validation are available from the Gene Expression Omnibus under accessions GSE207422 and GSE91061 and from the European Nucleotide Archive under accession PRJEB23709. Custom analysis code is available at https://github.com/JackFang666/NeuCCL3-nCIT-HPSCC/tree/main. All other data supporting the findings of this study are included in the article and its supplementary information files. Additional information is available from the corresponding author upon reasonable request.
