# Supplementary material for: CCL3+ Neutrophil Signature Predicts Response to Neoadjuvant Toripalimab plus Chemotherapy in Patients with Hypopharyngeal Squamous Cell Carcinoma: A Phase II Trial
Source: Clin Cancer Res. 2026 Mar 12;32(11):2166–82. doi: 10.1158/1078-0432.CCR-25-4096 (PMC13223550; doi:10.1158/1078-0432.CCR-25-4096)
Supplement: Supplementary File S1 — Supplementary: Clinical trial protocol [file ccr-25-4096_supplementary_file_s1_suppds1.pdf]

Supplementary: Clinical trial protocol

A Prospective, Single-Arm, Exploratory Clinical Study of Toripalimab Combined with Chemotherapy for Neoadjuvant Treatment of Head and Neck Squamous Cell Carcinoma

Clinical Study Protocol

Sponsor: Shandong Provincial ENT Hospital (Shandong Second Provincial General Hospital)

Principal Investigators:

Professor Zhenghua Lv

Professor Yumei Wei

Protocol Version/Date: Version 1.0 / December 06, 2022

Protocol Synopsis

Study Title	A Prospective, Single-Arm, Exploratory Clinical Study of Toripalimab Combined with Chemotherapy for Neoadjuvant Treatment of Head and Neck Squamous Cell Carcinoma
Sponsor	Shandong Provincial ENT Hospital (Shandong Second Provincial General Hospital)
Study Type	Investigator-Initiated Study
Study Population	Treatment-naïve patients with Stage III-IV hypopharyngeal cancer
Investigational Product	**Drug Name:** Toripalimab Injection **Trade Name:** TUOYI® **Specification:** 240 mg (6 mL)/vial **Dosage and Administration:** 240 mg administered once via intravenous infusion every 3 weeks
Sample Size	51 patients
Inclusion Criteria	1.Age 18-70 years.<br>2.ECOG performance status score ≤1.<br>3.Histopathologically confirmed hypopharyngeal or cervical esophageal squamous cell carcinoma.<br>4.Patients indicated for total laryngectomy or without surgical indications for their primary tumor, with Stage III-IV disease. Select T2 hypopharyngeal cancer patients may be enrolled if indicated for total laryngectomy.<br>5.No prior anti-tumor therapy, including radiotherapy,

	chemotherapy, immunotherapy, or biological therapy.<br>6. At least one measurable lesion per RECIST 1.1 criteria.<br>7.Life expectancy of at least 6 months.<br>8.No contraindications to chemotherapy or immunotherapy.<br>9.Adequate organ function: A. WBC≥3.0×10^9/L, ANC≥2.0×10^9/L B. Hb≥90g/L C. Platelets ≥100×10^9/L D. Serum albumin ≥2.8g/dL E. Total bilirubin ≤1.5×ULN; ALT and AST ≤3.0×ULN F. Serum creatinine ≤1.5×ULN or creatinine clearance >60mL/min<br>10.Fertile female subjects and male subjects with fertile female partners must agree to use a medically approved contraceptive method during the study and for at least 3 months after the last dose of toripalimab and 6 months after the last dose of chemotherapy or radiotherapy.<br>11.Voluntary participation with signed informed consent, good compliance, and willingness to cooperate with follow-up.
Exclusion Criteria	1. History of severe hypersensitivity to other monoclonal antibodies or any component of a PD-1 antibody.<br>2. Concurrent malignancies.<br>3. Severe psychiatric illness.<br>4. Severe heart disease or pulmonary dysfunction (cardiac/pulmonary function Grade 3 or lower).<br>5. Laboratory values not meeting inclusion criteria within 7 days

	prior to enrollment.<br>6. Systemic or local glucocorticoid therapy within 4 weeks prior to enrollment.<br>7. Comorbidities requiring long-term immunosuppressive drugs or immunosuppressive doses of corticosteroids.<br>8. Active tuberculosis (TB), currently receiving anti-TB therapy, or received anti-TB therapy within 1 year prior to screening.<br>9. HIV positive.<br>10. HBsAg positive with detectable HBV DNA (≥1000 cps/mL or IU/mL); HCV antibody positive.<br>11. Positive pregnancy test in women of childbearing potential; breastfeeding women.
Treatment Plan	1. Induction Chemotherapy: 2 cycles of TPF, TP, or PF regimen with prophylactic antiemetics, hepatoprotective agents, and hydration.<br>2. Toripalimab: During the induction phase, 240 mg administered via intravenous infusion once every 3 weeks for 2 cycles. Toripalimab is to be infused prior to chemotherapy, with an interval of at least 1 hour for the first cycle and at least 30 minutes for subsequent cycles.<br>3. Definitive Concurrent Chemoradiotherapy: Efficacy is assessed after 2 cycles of induction therapy. Patients with a primary tumor

	response of CR or PR (≥75% reduction) and a cervical lymph node response of CR, PR, or SD, or patients who decline surgery, will receive definitive radiotherapy and chemotherapy to the primary tumor and metastatic neck nodes. A third cycle of induction therapy (chemotherapy + toripalimab) is completed before radiotherapy. Efficacy is re-evaluated 1 and 3 months post-radiotherapy.<br>4. Surgical Treatment: Efficacy is assessed after 2 cycles of induction therapy. Patients with a primary tumor response of PR (<75% reduction), SD, or PD, and a cervical lymph node response of CR, PR, or SD, or patients with any primary tumor response but a lymph node response of PD, will undergo surgical treatment. The surgical plan will be determined by the head and neck surgery department after MDT discussion.
Primary Endpoint	Pathological Complete Response (pCR) Rate
Secondary Endpoints	Major Pathological Response (MPR) Rate, Objective Response Rate (ORR) after neoadjuvant therapy, 2-year Event-Free Survival (EFS) Rate, 2-year Overall Survival (OS) Rate, Safety
Statistical Analysis	Analysis will be performed using SPSS software. Continuous data will be described using medians, means, and standard deviations; categorical data will be described using counts and percentages. Survival data will be analyzed using the Kaplan-Meier method.

	Prognostic factors will be analyzed using the Log-rank test and Cox regression models.

TABLE OF CONTENTS

Protocol Synopsis 2
1.1 Induction Chemotherapy in Locally Advanced Head and Neck Squamous Cell Carcinoma 8
1.2 Advances in Immune Checkpoint Inhibitors for Head and Neck Squamous Cell Carcinoma 9
1.3 Advances in Toripalimab for Head and Neck Squamous Cell Carcinoma 13
2. Study Objectives 15
3. Study Design 16
3.1 Overall Study Design 16
3.2 Inclusion Criteria 16
3.3 Exclusion Criteria 18
3.4 Discontinuation of Study Treatment 19
4. Study Methods 20
4.1 Investigational Product 20
4.2 Dosing Regimen 20
4.3 Dose Adjustments and Treatment Delays 21
5. Clinical Study Procedure 25
5.1 Screening Period 25
5.2 Treatment Period 26
5.3 Follow-up Period 27
6. Study Endpoints and Evaluations 27
7. Management of Adverse Event 28
7.1 Definition and Classification of Adverse Events 29
7.2 Observation and Management of Adverse Events 30
7.3 Assessment of AE Causality to the Study Drug 31
7.4 Reporting of Adverse Events 32
8. Statistical Methods 33
8.1 Data Analysis Sets 33
8.2 Statistical Analysis Plan 34
9. Ethical Considerations 34
9.1 Investigator Responsibilities 35
9.2 Independent Ethics Committee (IEC)/Institutional Review Board (IRB) 35
9.3 Informed Consent 36
9.4 Confidentiality of Personal Data 36
10. Administrative Requirements 37
10.1 Protocol Amendments 37
10.2 Case Report Form (CRF) Completion 37
11. Study Timeline 37
12. References 38
13. Appendices 42
Appendix 1: ECOG Performance Status Scale 42
Appendix 2: RECIST 1.1 Tumor Evaluation Criteria 42

1.Background and Rationale

Head and neck squamous cell carcinoma (HNSCC) includes squamous cell carcinomas originating in the tongue, oral cavity, oropharynx, nasopharynx, and hypopharynx. According to the GLOBOCAN 2018 report, HNSCC ranks as the 9th most common malignancy worldwide, with 834,860 new cases and 431,131 deaths in 2018, accounting for approximately 4.6% of all new cancer cases and 4.6% of cancer-related deaths in that year[1]. According to the National Cancer Center's 2018 annual report on cancer incidence and mortality in China, in 2015 HNSCC was the 8th most common cancer nationwide, with 114,000 new cases and 58,000 deaths, accounting for about 3.0% and 2.5% of the national totals for new cancer cases and deaths, respectively[2].

1.1 Induction Chemotherapy in Locally Advanced Head and Neck Squamous Cell Carcinoma

For early-stage HNSCC, surgery and radiotherapy are the main treatment modalities. However, 70%-80% of patients present with locally advanced or advanced disease at initial diagnosis. In such patients, the 5-year survival rate after treatment with surgery or radiotherapy is only around 40% or lower, and survival drops significantly after recurrence. Induction chemotherapy refers to chemotherapy administered before surgery or radiotherapy. Because the tumor's blood supply is relatively intact prior to local treatment, induction chemotherapy facilitates the

delivery of chemotherapeutic drugs into tumor cells and creates favorable conditions for subsequent local therapy. Induction chemotherapy can improve the objective response rate (ORR), but it remains controversial whether this translates into a survival benefit. Theoretically, induction chemotherapy can shrink the tumor to create better conditions for subsequent local treatment, reduce the proliferative activity of tumor cells, decrease the chance of intraoperative tumor dissemination, improve the rate of local control, reduce the risk of distant metastases by treating micrometastatic disease, and thereby lower the incidence of distant metastasis.

Currently, the most commonly used induction chemotherapy regimen for HNSCC is TPF (docetaxel + cisplatin + 5-fluorouracil). The TAX323 and TAX324 studies showed that compared with the PF regimen (cisplatin + 5-fluorouracil), TPF induction chemotherapy significantly improved response rates and survival outcomes[3, 4]. Vermorken et al.[4] randomized 358 stage III–IV HNSCC patients without distant metastasis: 177 patients received TPF induction chemotherapy and 181 received PF induction chemotherapy. The overall response rate after induction therapy was 68% in the TPF group versus 54% in the PF group. With a median follow-up of 32.5 months, the median progression-free survival (PFS), overall survival (OS), and complete response (CR) rate were 11.0 vs. 8.2 months (P=0.007), 18.8 vs. 14.5 months (P=0.02), and 33.3% vs. 19.9% (P=0.004), respectively. The TPF group had a higher incidence of Grade 3-4 leukopenia than the PF group (76.9% vs. 52.5%), but a lower incidence of fatal toxic side effects (2.3% vs. 5.5%).

1.2 Advances in Immune Checkpoint Inhibitors for Head and Neck Squamous

Cell Carcinoma

Immune checkpoint inhibitor (ICI) therapy is a highly effective form of immunotherapy. Tumors can evade immune surveillance by expressing checkpoint ligands such as programmed death-1 (PD-1), programmed death ligand-1 (PD-L1), cytotoxic T-lymphocyte-associated protein 4 (CTLA-4), and others. Immune checkpoint inhibitors enhance the immune response against tumor cells by blocking these negative regulatory signals[5].

Owing to the success of immunotherapy in recurrent/metastatic head and neck squamous cell carcinoma (r/mHNSCC), multiple clinical guidelines have recommended ICIs as second-line or first-line treatments for this disease. Based on the results of CheckMate-141[6], KEYNOTE-012[7, 8], KEYNOTE-055[9], and KEYNOTE-040[10], nivolumab and pembrolizumab were shown to significantly prolong survival in r/mHNSCC patients who failed prior platinum-based chemotherapy and to improve their quality of life. Around 2016, the U.S. Food and Drug Administration (FDA) approved these agents for second-line treatment of r/mHNSCC. Subsequently, based on KEYNOTE-048[11], in 2019 the FDA approved pembrolizumab combined with chemotherapy as a first-line treatment for r/mHNSCC, and pembrolizumab monotherapy as a first-line treatment for PD-L1–positive r/mHNSCC patients. Similarly, the 2021 Chinese Society of Clinical Oncology (CSCO) Head and Neck Cancer Guidelines include pembrolizumab plus chemotherapy or pembrolizumab monotherapy (for CPS ≥1) as a Grade I expert recommendation for r/mHNSCC.

Given the reliable efficacy of ICIs in r/mHNSCC and the remarkable preliminary results of neoadjuvant immunotherapy in non-small cell lung cancer, melanoma, and other malignancies[12, 13], the application of ICIs before definitive treatment in HNSCC is highly anticipated. Neoadjuvant immunotherapy may have the following potential clinical benefits: (1) stratifying patients by their response to therapy; (2) reducing tumor burden and even down-staging the disease, thereby lowering local recurrence, distant metastasis, and mortality rates, reducing the extent of radical surgery, and better preserving organ function and appearance; (3) incurring fewer toxic side effects than induction chemotherapy, which reduces treatment-related adverse effects during definitive therapy and improves patient tolerability.

Wise-Draper et al.[14] conducted a phase II multicenter clinical trial of neoadjuvant and adjuvant pembrolizumab combined with standard adjuvant therapy in HNSCC. The study enrolled 80 patients with locally advanced HNSCC (T3-4 and/or N2-positive disease). All patients received a single dose of pembrolizumab 1–3 weeks before surgery. Based on post-surgical pathology results, patients then received either pembrolizumab plus radiotherapy or pembrolizumab plus chemoradiotherapy (pembrolizumab + radiotherapy + cisplatin). The treatment effect (TE) after neoadjuvant immunotherapy was evaluated by tumor regression and classified as no pathological response (NPR, <20%), partial pathological response (PPR, 20%-90%), or major pathological response (MPR, ≥90%).

Interim results from 66 patients showed that 51% of patients had high-risk pathological features post-surgery (positive margins or extranodal extension); the

1-year disease-free survival (DFS) rate for these high-risk patients was 66%. Patients with intermediate-risk pathology had a 1-year DFS rate of 91% [versus 69% in historical controls from RTOG 9501[6], P=0.05]. Among 70 patients evaluable for pathological response, 40 had NPR, 27 had PPR, and 3 had MPR. Notably, patients who achieved a pathological response (PPR + MPR) had a significantly higher 1-year DFS compared to those with NPR (100% vs. 57%, HR=0.18). These findings suggest that neoadjuvant plus adjuvant pembrolizumab can improve DFS in intermediate-risk patients, and that patients achieving a pathological response after neoadjuvant immunotherapy have a better 1-year DFS.

At the 2021 ASCO meeting, Uppaluri et al.[15] reported results of another phase II study in which two cycles of neoadjuvant pembrolizumab were given to HPV-negative, resectable locally advanced HNSCC patients. Among 25 evaluable patients, the pathological treatment response grade 2 (pTR-2) rate was 44%, and 4 patients achieved a pathological complete response (pCR). In a separate phase II trial, Zinner et al.[16] evaluated a neoadjuvant regimen of paclitaxel + carboplatin + nivolumab for locally advanced HNSCC. Preliminary results from 27 patients showed that 10 patients experienced Grade 3 treatment-related adverse events (TRAEs), 4 patients had Grade 3–4 neutropenia, and 6 patients required dose adjustments due to side effects. Postoperative pathology revealed that 11 of 26 (42%) patients achieved pCR at the primary site (with 1 patient's primary tumor not evaluable), and the combined MPR + pCR rate was 69% (18/26). Except for one patient who died from rapid recurrence, none of the other patients had relapsed after a median follow-up of

13 months. Neoadjuvant immunotherapy alone or in combination with chemotherapy has thus demonstrated encouraging efficacy and safety in locally advanced HNSCC, warranting further investigation.

1.3 Advances in Toripalimab for Head and Neck Squamous Cell Carcinoma

Toripalimab injection, developed by Shanghai Junshi Biosciences Co., Ltd., is a novel recombinant humanized (97% human) anti-PD-1 monoclonal antibody of the IgG4/kappa subtype (Chinese patent No. CN104250302B; PCT Publication No. WO2014/206107A1). Toripalimab binds to PD-1 and blocks its interaction with PD-L1 and PD-L2 (also known as B7-DC), thereby activating cytotoxic T lymphocytes and inhibiting tumor growth[17].

In 2018, toripalimab was approved by the China National Medical Products Administration (NMPA) and has since been approved for six indications in China, including:

1. treatment of unresectable or metastatic melanoma after failure of systemic therapy;

2. treatment of recurrent/metastatic nasopharyngeal carcinoma in patients who have failed at least second-line systemic therapy;

3. treatment of locally advanced or metastatic urothelial carcinoma that has progressed following platinum-based chemotherapy (including progression within 12 months of neoadjuvant or adjuvant chemotherapy);

4. first-line treatment of locally recurrent or metastatic nasopharyngeal

carcinoma in combination with cisplatin and gemcitabine;

5. first-line treatment of unresectable locally advanced, recurrent, or metastatic esophageal squamous cell carcinoma (ESCC) in combination with paclitaxel and cisplatin;

6. first-line treatment of epidermal growth factor receptor (EGFR) mutation-negative, anaplastic lymphoma kinase (ALK)-negative, unresectable locally advanced or metastatic non-squamous non-small cell lung cancer (NSCLC) in combination with pemetrexed and a platinum-based chemotherapy.

So far, no domestically developed PD-1 inhibitor has obtained an approved indication for HNSCC. However, toripalimab has been evaluated in clinical studies in the HNSCC field and has shown promising clinical efficacy. At ASCO 2022, an Ib/II study of toripalimab combined with cetuximab in platinum-refractory r/mHNSCC patients was reported[18]. Results from 12 patients indicated an objective response rate (ORR) of 50%, a disease control rate (DCR) of 100%, and a median duration of response (DOR) of 5.1 months. The efficacy was encouraging and the safety profile was tolerable, with no observed ≥ Grade 3 TRAEs or immune-related adverse events (irAEs).

The NeoTGP01 study[19] explored the safety and efficacy of neoadjuvant toripalimab combined with GP (gemcitabine + cisplatin) in resectable HNSCC patients. A total of 23 eligible patients were enrolled, and the incidences of Grade 1, 2, 3, and 4 TRAEs were 43.5%, 34.8%, 13.0%, and 8.7%, respectively. The most common TRAEs were decreased appetite and anorexia (39.1%), asthenia and fatigue

(26.0%), nausea (26.0%), and vomiting (26.0%). Grade 3–4 TRAEs included neutropenia, fatigue, hyperglycemia, nausea and vomiting, decreased appetite, rash, and diarrhea. Importantly, no treatment-related surgical delays were observed. In terms of efficacy, the radiologic response rate was 5.0% complete response (CR), 40.0% partial response (PR), and 55.0% stable disease (SD). Eighteen patients proceeded to successful surgical resection, with an R0 resection rate of 100%. Regarding pathological response, 3 patients (16.7%) achieved pCR at the primary tumor site, 5 patients (27.8%) achieved MPR (with 2 cases being near pCR), 3 patients (16.7%) achieved PPR, and 7 patients (38.8%) had NPR. This study demonstrated a favorable safety profile and substantial pCR/MPR rates with two cycles of neoadjuvant toripalimab plus GP in resectable HNSCC patients.

Based on the above evidence, this study will incorporate the PD-1 inhibitor toripalimab into standard induction chemotherapy regimens (TPF, PF, or TP) to investigate the efficacy and safety of toripalimab combined with chemotherapy as a neoadjuvant treatment for head and neck squamous cell carcinoma. The trial will mainly enroll patients with hypopharyngeal carcinoma and cervical esophageal carcinoma, in an effort to provide a new neoadjuvant treatment option for patients with locally advanced HNSCC.

2. Study Objectives

To evaluate the efficacy and safety of toripalimab combined with chemotherapy

for the neoadjuvant treatment of head and neck squamous cell carcinoma.

Primary Endpoint: Pathological Complete Response (pCR) rate.

Secondary Endpoints: Major Pathological Response (MPR) rate, overall Objective Response Rate (ORR) after neoadjuvant therapy, 2-year Event-Free Survival (EFS) rate, 2-year Overall Survival (OS) rate, and safety.

3. Study Design

3.1 Overall Study Design

This is a prospective, single-arm, exploratory clinical study designed to evaluate the efficacy and safety of toripalimab combined with chemotherapy as neoadjuvant treatment for head and neck squamous cell carcinoma.

Sample Size Calculation: This is a single-arm trial with the pathological complete response (pCR) rate as the primary endpoint. Based on historical data, the pCR rate for neoadjuvant induction chemotherapy is approximately 27%. This study anticipates an improvement in the pCR rate to 47%. Using PASS software, with a two-sided test, an alpha of 0.05, and 80% power, the required sample size is calculated to be 42 patients. Accounting for a potential dropout rate of 18%, the total number of patients to be enrolled is 51.

3.2 Inclusion Criteria

1.Age 18-70 years.

2.Eastern Cooperative Oncology Group (ECOG) performance status score ≤1.

3.Histopathologically confirmed squamous cell carcinoma of the hypopharynx or cervical esophagus.

4.Treatment-naïve patients for whom the primary surgical approach is total laryngectomy or who have no surgical indications, with Stage III-IV disease. Select patients with T2 hypopharyngeal cancer may be enrolled if they are candidates for total laryngectomy.

5.No prior anti-tumor therapy, including radiotherapy, chemotherapy, immunotherapy, or biological therapy.

6.At least one measurable lesion according to RECIST 1.1 criteria.

7.Life expectancy of at least 6 months.

8.No contraindications to chemotherapy or immunotherapy.

9.Adequate organ function as defined by the following laboratory values:

A. White blood cell (WBC) count $\geq 3.0\times 10^9$/L, Absolute neutrophil count (ANC) $\geq 2.0\times 10^9$/L.

B. Hemoglobin (Hb) ≥90g/L.

C. Platelet count $\geq 100\times 10^9$/L.

D. Serum albumin ≥2.8g/dL.

E. Total bilirubin ≤1.5× upper limit of normal (ULN); Alanine aminotransferase (ALT) and Aspartate aminotransferase (AST) ≤3.0×ULN.

F. Serum creatinine ≤1.5×ULN or creatinine clearance >60mL/min.

10.Female subjects of childbearing potential and male subjects with partners of

childbearing potential must agree to use a medically approved method of contraception (e.g., intrauterine device, oral contraceptives, or condoms) during the study treatment period and for at least 3 months after the last dose of toripalimab and at least 6 months after the last dose of chemotherapy or radiotherapy.

11.The subject voluntarily agrees to participate in the study, signs the informed consent form, has good compliance, and is willing to cooperate with follow-up procedures.

3.3 Exclusion Criteria

1.History of severe hypersensitivity to other monoclonal antibodies or any component of a PD-1 antibody.

2.Presence of other concurrent malignancies.

3.Concurrent severe psychiatric illness.

4.Severe heart disease or pulmonary dysfunction; cardiac or pulmonary function of Grade 3 or lower.

5.Laboratory values not meeting the inclusion criteria within 7 days prior to enrollment.

6.Systemic or local glucocorticoid therapy within 4 weeks prior to enrollment.

7.Presence of comorbidities requiring long-term treatment with immunosuppressive drugs or systemic or local use of corticosteroids at immunosuppressive doses.

8.Patients with active tuberculosis (TB), currently receiving anti-TB therapy, or having received anti-TB therapy within 1 year prior to screening.

9.Positive for Human Immunodeficiency Virus (HIV).

10.Positive for Hepatitis B surface antigen (HBsAg) with detectable HBV DNA copy number (quantitative detection ≥1000 cps/mL); positive screening for chronic hepatitis C (HCV antibody positive).

11.Women of childbearing potential with a positive pregnancy test or who are breastfeeding.

3.4 Discontinuation of Study Treatment

A subject may be withdrawn from the study for any of the following reasons:

1.Occurrence of a serious safety issue during the trial that necessitates immediate termination.

2.Discovery of a major flaw in the study protocol that makes it difficult to evaluate the endpoints, or a significant deviation in the implementation of a well-designed protocol that makes further continuation untenable for endpoint evaluation.

3.The investigator requests termination (e.g., due to funding or administrative reasons).

4.An administrative body or ethics committee requests termination of the study.

5.The investigator believes that continued treatment may be detrimental to the subject's best interests.

If the investigator determines that a subject needs to be withdrawn based on the above criteria, or if a subject requests to withdraw for personal reasons, the study drug will be stopped, appropriate medical measures will be provided, and necessary

examinations (physical, laboratory, etc.) will be conducted. The completed sections of the CRF will be filled out, with the date and reason for discontinuation clearly noted.

4. Study Methods

4.1 Investigational Product

Investigational Drug: Toripalimab Injection, Specification: 240 mg (6 mL)/vial.

4.2 Dosing Regimen

1.Induction Chemotherapy Regimen: Two cycles of TPF, TP, or PF chemotherapy will be administered, along with prophylactic antiemetics, hepatoprotective agents, hydration, and other symptomatic treatments.

2.Toripalimab: During the induction chemotherapy phase, toripalimab 240 mg will be administered once via intravenous infusion every 3 weeks for a total of 2 cycles. The toripalimab infusion should precede chemotherapy administration by at least 1 hour for the first two cycles and by at least 30 minutes for subsequent cycles.

3.Definitive Concurrent Chemoradiotherapy: Efficacy will be assessed after 2 cycles of induction therapy with toripalimab and chemotherapy. Patients whose primary tumor shows a Complete Response (CR) or Partial Response (PR) (tumor shrinkage ≥75%) and whose cervical lymph nodes show CR, PR, or Stable Disease

(SD), or patients who decline surgery, will receive definitive radiotherapy and chemotherapy to the primary tumor and metastatic neck nodes. The second cycle of induction therapy (chemotherapy + toripalimab) should be completed before the start of radiotherapy. Efficacy will be re-evaluated 1 and 3 months after the completion of radiotherapy.

4.Surgical Treatment: Efficacy will be assessed after 2 cycles of induction therapy. Patients whose primary tumor shows a PR (tumor shrinkage <75%), SD, or Progressive Disease (PD), and whose cervical lymph nodes show CR, PR, or SD, or patients with any primary tumor response but PD in the cervical lymph nodes, will undergo surgical treatment. The surgical plan will be formulated by the head and neck surgery department following a Multidisciplinary Team (MDT) discussion.

4.3 Dose Adjustments and Treatment Delays

1.If a subject experiences severe toxicity, the investigator will determine the causality in relation to the immunotherapy and chemotherapy drugs. If the toxicity is clearly or likely attributable to one drug, the investigator may choose to reduce the dose of that drug. If the causality is uncertain, the investigator will determine the management strategy.

2.During the study, each chemotherapy drug is permitted a maximum of two dose adjustments, with each reduction being 25%. Once a dose is reduced, it cannot be increased in subsequent cycles.

3.The investigator may use supportive care measures to treat/prevent toxicities as an alternative to dose reduction in the next cycle if it is deemed in the subject's best interest.

4.Dose adjustment of chemotherapy drugs is permitted in the next cycle if the subject experiences any of the following after a dose:

A.ANC count $<0.5\times10^9$/L for 7 days.

B.Febrile neutropenia (FN).

C.ANC count between 0.5×10^9/L and 1.0×10^9/L with a platelet (PLT) count between 25×10^9/L and 50×10^9/L for 7 days.

D.PLT count $<25\times10^9$/L, or bleeding, or requiring transfusion.

E.≥ Grade 3 anemia.

F.≥ Grade 3 peripheral neuropathy or gastrointestinal toxicity.

G.Other Grade 3 or higher non-hematological toxicities.

5.Dose Adjustment Principles for PD-1 Antibody:

Immune-Related Adverse Reaction	Severity	Treatment Adjustment Plan
Immune-related pneumonitis	Grade 2 pneumonitis	Withhold treatment until symptoms resolve, radiological abnormalities improve, and corticosteroid therapy is complete
	Grade 3 or 4 pneumonitis	Permanently discontinue

Immune-related colitis	Grade 2 diarrhea or colitis	Withhold treatment until symptoms resolve and corticosteroid therapy (if required) is complete
	Grade 3 diarrhea or colitis	Withhold treatment until symptoms resolve and corticosteroid therapy is complete
	Grade 4 diarrhea or colitis	Permanently discontinue
Immune-related hepatitis	Grade 2 elevation of AST, ALT, or total bilirubin	Withhold treatment until laboratory values return to baseline and corticosteroid therapy (if required) is complete
	Grade 3 or 4 elevation of AST, ALT, or total bilirubin	Permanently discontinue
Immune-related nephritis and renal dysfunction	Grade 2 or 3 creatinine elevation	Withhold treatment until creatinine returns to baseline and corticosteroid therapy is complete
	Grade 4 creatinine elevation	Permanently discontinue
Immune-related endocrinopathies	Symptomatic Grade 2 or 3 hypothyroidism, hyperthyroidism, hypophysitis	Withhold treatment until symptoms resolve and corticosteroid therapy (if needed for acute

	Grade 2 adrenal insufficiency	inflammatory symptoms) is complete
	Grade 3 diabetes mellitus	While on hormone replacement therapy, treatment should be maintained as long as asymptomatic
	Grade 4 hypothyroidism<br>Grade 4 hyperthyroidism<br>Grade 4 hypophysitis<br>Grade 3 or 4 adrenal insufficiency<br>Grade 4 diabetes mellitus	Permanently discontinue
Immune-related dermatologic adverse reactions	Grade 3 rash	Withhold treatment until symptoms resolve and corticosteroid therapy is complete
	Grade 4 rash	Permanently discontinue
	Stevens-Johnson Syndrome (SJS) or Toxic Epidermal Necrolysis (TEN)	Permanently discontinue
other immune-related adverse reactions	Grade 3 (first occurrence)	Withhold treatment
	Grade 3 myocarditis	Permanently discontinue
	Grade 4 or recurrent Grade 3: persistent Grade 2 or 3	Permanently discontinue

	reaction despite treatment adjustment, corticosteroid dose cannot be reduced to 10 mg prednisone or equivalent per day	

Immunotherapy should be permanently discontinued in the following situations:

Grade 4 or recurrent Grade 3 adverse reactions;Persistent Grade 2 or 3 adverse reactions despite adjustments.

5. Clinical Study Procedure

A medical history will be obtained at the screening visit, which must include the date of HNSCC diagnosis and the histological or cytological record of the malignancy. The study is divided into three phases: screening, treatment, and follow-up.

If a subject experiences a treatment delay due to adverse events or other reasons, treatment will resume once the subject meets the criteria for dosing. Subsequent treatment cycles and visit schedules will be postponed according to the length of the delay.

5.1 Screening Period

This period includes a pre-enrollment physical examination (including cranial nerve examination), complete blood count (CBC), comprehensive metabolic panel, coagulation function tests, tumor markers (SCC, CYFRA21-1), urinalysis with

microscopy, stool routine with occult blood test, urine pregnancy test, thyroid function tests, CK and CK-MB, infection screening, histopathological examination, PD-L1 protein expression analysis, contrast-enhanced MRI/CT of the head and neck (or non-contrast scans if contraindicated), chest CT, abdominal ultrasound or CT, and cervical lymph node ultrasound, and electrocardiogram (ECG). All examinations must be completed within 4 weeks prior to the subject's entry into the study. A detailed medical history, including past illnesses, and a full physical examination (including ECOG performance status, height, and weight) and a specialized tumor examination must be completed before enrollment. Pre-treatment tumor lesion sizes will be measured using imaging studies and recorded. The study, including the treatment plan and procedures, will be explained to the subject and their family.

5.2 Treatment Period

Before each treatment cycle, a physical examination will be conducted, including ECOG performance status, blood pressure, heart rhythm, heart rate, temperature, pulse, and respiration. Laboratory tests including CBC, comprehensive metabolic panel, coagulation function, tumor markers (SCC, CYFRA21-1), urinalysis with microscopy, stool routine with occult blood, thyroid function, CK and CK-MB, and an ECG will be performed. Continuous ECG monitoring will be provided during drug administration and for 2 hours after completion if no discomfort occurs. If adverse symptoms arise, ECG monitoring will continue until symptoms have ceased for more than 12 hours. The subject's condition and any adverse reactions related to the

medication (e.g., allergic reactions, injection site reactions, chest tightness, chest pain, dizziness, headache, fatigue, gastrointestinal reactions) will be closely monitored and recorded. Symptomatic and supportive care will be provided for any clinical symptoms until they resolve or disappear.

Treatment will be administered according to the protocol. After 3 cycles of induction chemotherapy plus toripalimab, an efficacy evaluation will be performed. This will include routine physical examination, ECOG performance status scoring, and all laboratory and imaging tests as performed during screening. If a subject's symptoms worsen during treatment, an early efficacy evaluation may be conducted to determine if it is due to disease progression or other special circumstances.

5.3 Follow-up Period

Subjects who undergo surgery will enter the follow-up period on the day after their operation. The adjuvant treatment plan post-surgery will be determined by the investigator based on clinical experience. Subjects who receive concurrent chemoradiotherapy will enter the follow-up period after completing the neoadjuvant induction therapy. Follow-up will be conducted every 3 months for the first 2 years, every 6 months for years 3-5, and annually thereafter.

6. Study Endpoints and Evaluations

1. Primary Endpoint

Pathological Complete Response (pCR) Rate: Defined as 0% residual viable tumor cells in the surgical specimen.

2. Secondary Endpoints

Major Pathological Response (MPR) Rate: Defined as ≤10% residual viable tumor cells in the surgical specimen.

Overall Response Rate (ORR): The proportion of patients with a Complete Response (CR) or Partial Response (PR) among all evaluable cases, calculated as (CR+PR)/Total Evaluable Cases. Tumor response will be assessed using the Response Evaluation Criteria in Solid Tumors (RECIST 1.1). Subjects must have measurable tumor lesions at baseline. Response categories are CR, PR, Stable Disease (SD), and Progressive Disease (PD).

Event-Free Survival (EFS): The time from randomization to the first occurrence of any event, including disease progression preventing surgery, local or distant recurrence, or death from any cause.

Overall Survival (OS): The time from the date of randomization to death from any cause.

Safety Analysis: Assessed according to the National Cancer Institute Common Terminology Criteria for Adverse Events (NCI-CTCAE) v5.0. A list of all deaths, all SAEs, and all drug-related AEs will be generated.

7. Management of Adverse Event

The timely and accurate reporting and analysis of safety information in clinical research are crucial for protecting subjects, investigators, and sponsors, and are mandated by regulatory agencies. The sponsor has established standard operating procedures in accordance with relevant regulatory requirements to ensure the proper reporting of safety information. All clinical studies conducted by the sponsor or its affiliates will adhere to these procedures.

7.1 Definition and Classification of Adverse Events

1.Adverse Event (AE): Any untoward medical occurrence in a subject from the time of signing the informed consent form until the end of the follow-up period, regardless of its causal relationship with the investigational drug. This includes new adverse events, exacerbations of pre-existing conditions in severity or frequency, and abnormal diagnostic findings, including clinically significant laboratory abnormalities. All AEs during the trial will be accurately recorded, including their onset time, severity, duration, measures taken, and outcome.

2.Serious Adverse Event (SAE): SAEs should be collected from the time a subject signs the informed consent form. If an SAE occurs after signing the consent but before receiving the study drug, it does not need to be collected unless the investigator believes it may be caused by a protocol-mandated procedure. An AE is considered serious if it results in any of the following outcomes:

A.Death

B.A life-threatening situation

C.Requires inpatient hospitalization or prolongation of existing hospitalization

D.Results in persistent or significant disability/incapacity

E.Is a congenital anomaly/birth defect

Is an important medical event that, based on appropriate medical judgment, may jeopardize the subject or require medical or surgical intervention to prevent one of the outcomes listed above.

7.2 Observation and Management of Adverse Events

Observation and Recording: The investigator must diligently observe any AEs occurring during the clinical study and ask the subject to report any changes in their condition after medication, avoiding leading questions. While observing efficacy, attention must be paid to adverse reactions or unexpected toxic effects (including symptoms, signs, and laboratory findings). All AEs, whether related to the investigational drug or not, must be recorded in detail in the CRF, including onset time, symptoms, signs, severity, duration, laboratory values, management, course, outcome, and follow-up time. Concomitant medications must also be recorded in detail to facilitate the analysis of the AE's relationship with the investigational drug. The record must be signed and dated.

Medical Management: Upon detection of an adverse reaction, the investigator may take necessary measures based on the subject's condition, such as dose adjustment or temporary interruption of the drug, and decide whether to terminate the

trial. In the event of an SAE, the investigational site must immediately take necessary actions to protect the subject's safety.

If a subject becomes pregnant during the study, the research staff must submit an initial pregnancy report to the investigator via a pregnancy notification form within 24 hours of learning of the pregnancy. Abnormal pregnancy outcomes (e.g., spontaneous abortion, stillbirth, and congenital anomalies) are considered SAEs and must be reported using the SAE report form. Subjects who become pregnant during the study must discontinue further study treatment.

Follow-up of SAEs: If an SAE occurs that is unrelated to the investigational drug, causes no serious harm to the subject, and has no significant impact on the primary study endpoint, and the subject can adhere to the follow-up schedule, the trial may continue. All AEs should be followed until they resolve or return to baseline to ensure the subject's safety. The mode of follow-up (e.g., inpatient, outpatient, home visit, phone call, correspondence) can be chosen based on the severity of the adverse reaction.

7.3 Assessment of AE Causality to the Study Drug

The severity of AEs must be recorded and graded according to a toxicity grading scale (NCI-CTCAE v5.0). The relationship of the AE to the study drug should be assessed according to the following definitions:

1.Unrelated: There is evidence that the cause of the AE is not the study drug (e.g., pre-existing condition, underlying disease, concurrent illness, or concomitant

medication).

2.Unlikely: The event does not have a reasonable temporal relationship to the use of the study drug and could be caused by many other factors (e.g., clinical condition, other treatments, or concomitant medications).

3.Possible: There is a temporal relationship between the onset of the event and the administration of the study drug, but it cannot be well explained by the subject's clinical condition or concomitant treatments. It also seems plausible based on the known therapeutic effects and pharmacology of the drug. The event resolves or disappears upon drug discontinuation or dose reduction but reappears upon re-challenge. Note: It must be emphasized that lack of efficacy should not be considered a cause of an AE.

4.Related: The event has a reasonable temporal relationship to the use of the study drug, and the study drug is known or suspected to cause such an event. The event is alleviated or disappears upon discontinuation of the study drug and reappears upon re-administration.

5.Not Assessable: Insufficient information to determine causality.

These criteria, in addition to good clinical judgment, must be used as a guide for determining causality. If an event is considered unrelated to the study drug treatment, another explanation must be provided.

7.4 Reporting of Adverse Events

During the clinical study, the investigator must truthfully and in detail fill out the

adverse event record form for any AE, regardless of its relationship to the study medication. This includes recording the clinical manifestations, onset time, severity, duration, measures taken, and outcome. Concomitant medications should also be recorded in detail to facilitate the analysis of the AE's relationship to the study drug.

Any SAE occurring during the study must be reported by the study center staff to the investigator and the Pharmacovigilance (PV) department of Shanghai Junshi Biosciences Co., Ltd. within 1 day of becoming aware of the event (immediately, but no later than the end of the next working day). The SAE reporting email for the PV department is: CT_saereporting@junshipharma.com. Simultaneously, the ethics committee of the clinical trial institution must be notified by phone/fax within 24 hours, and a written report must be submitted by the investigator to the hospital's adverse reaction monitoring center within 72 hours. The sponsor should promptly investigate the SAE with the investigator and take necessary measures to ensure the safety and rights of the subjects.

The investigator must complete the "Serious Adverse Event Report Form." The original data should record when, how, and to whom the SAE was reported. The sponsor will ensure that all legally required reporting procedures are met.

8. Statistical Methods

8.1 Data Analysis Sets

1.Full Analysis Set (FAS): Based on the intention-to-treat (ITT) principle, this

set will include all enrolled subjects as much as possible. Subjects who did not receive the study drug, violated major inclusion criteria, or have no post-dose observation data may be excluded from the FAS. The FAS will be the primary population for analyzing baseline characteristics and efficacy based on imaging.

2.Per Protocol Set (PPS): This set includes subjects from the FAS who had no major protocol deviations, showed good compliance, and for whom the primary endpoint data are available. The PPS will be the primary population for evaluating efficacy based on pathological response.

3.Safety Analysis Set (SAS): This set includes all enrolled subjects who received at least one dose of neoadjuvant therapy and have post-dose safety data. This dataset will be used for safety analysis.

8.2 Statistical Analysis Plan

1. Analysis will be performed using SPSS software (version 20.0 or higher).

2. All statistical tests will be two-sided, and a P-value less than or equal to 0.05 will be considered statistically significant.

3. Continuous data will be described using medians, means, and standard deviations. Categorical data will be described using counts and percentages. Survival-related data will be analyzed using the Kaplan-Meier method, and prognostic factors will be analyzed using the Log-rank test and Cox regression models.

9. Ethical Considerations

9.1 Investigator Responsibilities

The investigator is responsible for ensuring that the clinical study is conducted in accordance with the study protocol, current Good Clinical Practice (GCP) guidelines, and relevant regulations of the China National Medical Products Administration (NMPA).

9.2 Independent Ethics Committee (IEC)/Institutional Review Board (IRB)

Before initiating the study, the investigator must submit the following documents to the IEC/IRB:

1. The final version of the study protocol (and any amendments).
2. The sponsor-approved informed consent form and any other written information to be provided to subjects.
3. The Investigator's Brochure.
4. Materials used for subject recruitment.
5. Any other documents required by the IEC/IRB.

The study may only begin after the IEC/IRB has fully approved the study protocol, informed consent form, and recruitment materials, and the sponsor has received a copy of the IEC/IRB approval letter. This letter must specify the approved study title (and number), the names and version numbers of the approved documents, and the date of approval.

9.3 Informed Consent

Each subject (or their legal representative) must provide written informed consent after being fully informed about the purpose and content of the study. This consent must be signed and dated before any study-related procedures are performed. The informed consent process must comply with the Declaration of Helsinki, current GCP guidelines, and relevant regulations. The investigator or a designated team member will explain the study's purpose, methods, potential benefits, potential risks, and any discomforts. Subjects will be informed that their participation is voluntary and that they may withdraw at any time without penalty or loss of benefits to which they are otherwise entitled. The subject's identity will be kept confidential but may be reviewed by regulatory authorities and the sponsor as permitted by law.

9.4 Confidentiality of Personal Data

This study will only collect and process data that are essential for researching the efficacy, safety, quality, and application of the drug. The confidentiality of this data will be fully ensured, and all relevant privacy protection laws and regulations will be followed. The sponsor will ensure that data collection is fair and lawful, for specific and legitimate purposes, and that the data are adequate, relevant, and not excessive in relation to the research objectives. Appropriate technical and organizational measures will be taken to protect personal data from unauthorized access, disclosure, accidental or unlawful destruction, accidental loss, and alteration.

10. Administrative Requirements

10.1 Protocol Amendments

Neither the investigator nor the sponsor may amend the protocol without the other's consent. Any modification that affects the conduct of the study, patient benefit, study objectives, design, patient numbers, or procedures must be submitted as a formal protocol amendment. Such amendments must be agreed upon by the investigator, sponsor, and ethics committee before implementation.

10.2 Case Report Form (CRF) Completion

The CRF provided by the sponsor must be completed in blue or black ink. All required information must be filled in the designated spaces. If information is not applicable, "NA" should be entered; if a test was not done, "ND" should be entered; if information is not available, "NAV" should be entered. No fields should be left blank. Any corrections must be made by drawing a single line through the error, writing the correct information nearby, and initialing and dating the change. The CRF should not contain the patient's name but should use initials and the patient identification number. The investigator must sign and date the CRF.

11. Study Timeline

Dec 2022 - Jan 2023: Finalize study protocol and related documents.

Jan 2023 - Feb 2023: Obtain ethics approval and sign trial agreements.

Mar 2023 - Mar 2024: Complete patient enrollment and treatment.

Mar 2024 - Mar 2026: Follow-up of the last enrolled subject is completed.

Mar 2026 - May 2026: Organize trial data, complete study summary and report.

12. References

1. Bray F, Ferlay J, Soerjomataram I, Siegel RL, Torre LA, Jemal A. Global cancer statistics 2018: GLOBOCAN estimates of incidence and mortality worldwide for 36 cancers in 185 countries. CA Cancer J Clin. 2018;68(6):394-424.

2. Chen W, Zheng R, Baade PD, Zhang S, Zeng H, Bray F, et al. Cancer statistics in China, 2015. CA Cancer J Clin. 2016;66(2):115-32.

3. Posner MR, Hershock DM, Blajman CR, Mickiewicz E, Winquist E, Gorbounova V, et al. Cisplatin and fluorouracil alone or with docetaxel in head and neck cancer. N Engl J Med. 2007;357(17):1705-15.

4. Vermorken JB, Remenar E, van Herpen C, Gorlia T, Mesia R, Degardin M, et al. Cisplatin, fluorouracil, and docetaxel in unresectable head and neck cancer. N Engl J Med. 2007;357(17):1695-704.

5. Solomon B, Young RJ, Rischin D. Head and neck squamous cell carcinoma: Genomics and emerging biomarkers for immunomodulatory cancer treatments. Semin Cancer Biol. 2018;52(Pt 2):228-40.

6. Ferris RL, Blumenschein G, Jr., Fayette J, Guigay J, Colevas AD, Licitra L, et al. Nivolumab vs investigator's choice in recurrent or metastatic squamous cell carcinoma of the head and neck: 2-year long-term survival update of CheckMate 141 with analyses by tumor PD-L1 expression. Oral Oncol. 2018;81:45-51.

7. Mehra R, Seiwert TY, Gupta S, Weiss J, Gluck I, Eder JP, et al. Efficacy and safety of pembrolizumab in recurrent/metastatic head and neck squamous cell carcinoma: pooled analyses after long-term follow-up in KEYNOTE-012. Br J Cancer. 2018;119(2):153-9.

8. Seiwert TY, Burtness B, Mehra R, Weiss J, Berger R, Eder JP, et al. Safety and clinical activity of pembrolizumab for treatment of recurrent or metastatic squamous cell carcinoma of the head and neck (KEYNOTE-012): an open-label, multicentre, phase 1b trial. Lancet Oncol. 2016;17(7):956-65.

9. Bauml J, Seiwert TY, Pfister DG, Worden F, Liu SV, Gilbert J, et al. Pembrolizumab for Platinum- and Cetuximab-Refractory Head and Neck Cancer: Results From a Single-Arm, Phase II Study. J Clin Oncol. 2017;35(14):1542-9.

10. Harrington KJ, Cohen EEW, Soulières D, Dinis J, Licitra L, Ahn MJ, et al. Pembrolizumab versus methotrexate, docetaxel, or cetuximab in recurrent or metastatic head and neck squamous cell carcinoma (KEYNOTE-040): Subgroup analysis by pattern of disease recurrence. Oral Oncol. 2023;147:106587.

11. Burtness B, Harrington KJ, Greil R, Soulières D, Tahara M, de Castro G, Jr., et al. Pembrolizumab alone or with chemotherapy versus cetuximab with chemotherapy for recurrent or metastatic squamous cell carcinoma of the head and neck (KEYNOTE-048): a randomised, open-label, phase 3 study. Lancet. 2019;394(10212):1915-28.

12. Topalian SL, Taube JM, Pardoll DM. Neoadjuvant checkpoint blockade for cancer immunotherapy. Science. 2020;367(6477).

13. Forde PM, Chaft JE, Smith KN, Anagnostou V, Cottrell TR, Hellmann MD, et al. Neoadjuvant PD-1 Blockade in Resectable Lung Cancer. N Engl J Med. 2018;378(21):1976-86.

14. Tang AL, O'Neil T, McDermott S, Tripathi S, Tikhtman R, Mark JR, et al. Association of Neoadjuvant Pembrolizumab for Oral Cavity Squamous Cell Carcinoma With Adverse Events After Surgery in Treatment-Naive Patients. JAMA Otolaryngol Head Neck Surg. 2022;148(10):935-9.

15. Uppaluri R, Chernock R, Mansour M, Jackson R, Rich J, Pipkorn P, et al. Enhanced pathologic tumor response with two cycles of neoadjuvant pembrolizumab in surgically resectable, locally advanced HPV-negative head and neck squamous cell carcinoma (HNSCC). Journal of Clinical Oncology.39(15_suppl):6008-.
16. Zinner R, Johnson JM, Tuluc M, Curry JM, Luginbuhl A, Fundakowski CC, et al. Neoadjuvant nivolumab (N) plus weekly carboplatin (C) and paclitaxel (P) in resectable locally advanced head and neck cancer. Journal of Clinical Oncology.38(15_suppl):6583-.
17. Xu R-h, Wang F, Shi J, Feng JF, Shen L, Yang S, et al. Recombinant humanized anti-PD-1 monoclonal antibody (JS001) as salvage treatment for advanced esophageal squamous cell carcinoma: Preliminary results of an open-label, multi-cohort, phase Ib/II clinical study. Journal of Clinical Oncology.36(4_suppl):116-.
18. Guo Y, Li Z, Chen W, Fang M, Liu Z, null n. Preliminary safety and efficacy of toripalimab combined with cetuximab in platinum-refractory recurrent or metastatic head and neck squamous cell carcinoma (R/M-HNSCC): A phase Ib/II clinical trial. Journal of Clinical Oncology.40(16_suppl):6022-.
19. Huang X, Liu Q, Zhong G, Peng Y, Liu Y, Liang L, et al. Neoadjuvant toripalimab combined with gemcitabine and cisplatin in resectable locally advanced head and neck squamous cell carcinoma (NeoTGP01): An open label, single-arm, phase Ib clinical trial. J Exp Clin Cancer Res. 2022;41(1):300.

13. Appendices

Appendix 1: ECOG Performance Status Scale

Score	Description
0	Fully active, able to carry on all pre-disease activities without restriction.
1	Restricted in physically strenuous activity but ambulatory; able to carry out light work such as light housework or office work.
2	Ambulatory and capable of all self-care but unable to carry out any work activities; up and about more than 50 % of waking hours.
3	Capable of only limited self-care; confined to bed or chair more than 50 % of waking hours.
4	Completely disabled; cannot carry on any self-care; totally confined to bed or chair.
5	Dead.

Appendix 2: RECIST 1.1 Tumor Evaluation Criteria

The Response Evaluation Criteria in Solid Tumours (RECIST 1.1) define how to classify and measure lesions at baseline and during follow-up. To determine objective response, the same imaging modality and measurement methodology should be used throughout the study.

1. Tumour measurability at baseline

1.1 Definitions

At baseline, lesions are categorised as **measurable** or **non-measurable**.

1.1.1 Measurable lesions

Measurable lesions have at least one dimension that can be accurately measured. The minimum sizes are:

Solid tumours: the longest diameter must be ≥ 10 mm on a CT scan with slice thickness ≤ 5 mm;

Clinical measurement: longest diameter ≥ 10 mm when using callipers or other measuring device; lesions that cannot be accurately measured using clinical instruments should be considered non-measurable;

Chest X-ray: longest diameter ≥ 20 mm;

Pathologically enlarged lymph nodes: short-axis diameter ≥ 15 mm on CT (preferred slice thickness ≤ 5 mm). At baseline and follow-up, only the short-axis diameter should be recorded.

1.1.2 Non-measurable lesions

Non-measurable lesions include small lesions (longest diameter < 10 mm or pathologic lymph nodes with short-axis ≥ 10 mm but < 15 mm) and truly unmeasurable lesions such as leptomeningeal disease, ascites, pleural or pericardial

effusion, inflammatory breast cancer, lymphangitic spread to the skin or lung, abdominal masses that cannot be accurately measured by imaging, and cystic lesions.

1.1.3 Special considerations for certain lesions

Bone lesions: bone scan, PET or plain radiographs are not suitable for measuring bone lesions but may confirm their presence or resolution. Lytic or mixed lytic/sclerotic lesions with a measurable soft-tissue component can be considered measurable if assessed by CT or MRI. Purely sclerotic lesions are non-measurable.

Cystic lesions: lesions that meet radiological criteria for simple cysts should not be considered malignant and are neither measurable nor non-measurable. Cystic metastases that meet measurability criteria may be selected as measurable lesions; however, if both cystic and non-cystic lesions are present in the same patient, non-cystic lesions should be preferred as target lesions.

Lesions previously treated with local therapy: lesions located in previously irradiated or locally treated areas are generally considered non-measurable unless there is unequivocal progression. The protocol should describe the conditions under which such lesions may be regarded as measurable.

1.2 Measurement methods

1.2.1 Recording of measurements

All measurements should be recorded in metric units. Baseline assessments

should be performed as close as possible to the start of treatment and within 28 days (4 weeks) before the first dose.

1.2.2 Assessment techniques

The same imaging technique and measurement methodology must be used for baseline and follow-up assessments. Apart from lesions that cannot be imaged and can only be assessed clinically, all lesions should be evaluated by imaging.

Clinical lesions: only superficial lesions with a diameter ≥ 10 mm are considered measurable (e.g. skin nodules). Colour photographs containing a ruler are recommended to document such lesions. When a lesion can be assessed both clinically and radiologically, radiologic evaluation is preferred because it is more objective and allows central review.

Chest X-ray: when tumour progression is an important study endpoint, chest CT is preferred because it is more sensitive, especially for detecting new lesions. Chest X-ray should only be used when measurable lesions have clearly defined borders and adequate lung expansion.

CT or MRI: CT with slice thickness ≤ 5 mm is the most reproducible method for assessing response. If slice thickness is > 5 mm, the minimum measurable lesion size should be twice the slice thickness. MRI is acceptable in certain circumstances (e.g. whole-body scanning).

Ultrasound: ultrasound should not be used to measure lesions because its operator dependence precludes reproducibility. If ultrasound suggests a new lesion, CT or MRI should be used to confirm. When radiation exposure is a concern, MRI may substitute for CT.

Endoscopy or laparoscopy: not recommended for objective tumour assessment, but may be used to obtain biopsy specimens to confirm a complete response or recurrence.

Cytological or histological techniques: under protocol-specified conditions, these techniques may be used to verify partial or complete responses (e.g. to differentiate residual benign tissue from malignant components). When effusions may be a side effect of therapy (such as with taxanes or angiogenesis inhibitors) and measurable disease meets criteria for response or stable disease, cytology can distinguish response/stable disease from progression.

2. Evaluation of tumour response

2.1 Assessment of the entire tumour burden and measurable lesions

To evaluate objective response or future progression, it is necessary to establish the total tumour burden at baseline. In studies where objective response is a primary endpoint, only measurable lesions should be selected as target lesions; non-measurable lesions should be recorded and followed qualitatively as non-target lesions.

2.2 Baseline documentation of target and non-target lesions

At baseline, a maximum of five measurable lesions per organ and no more than ten lesions in total should be selected as target lesions, representing all involved organs. Each target lesion should be measured and recorded individually. These selections should be revisited at baseline to ensure they represent the overall disease burden.

All other lesions, including measurable lesions not chosen as targets and all non-measurable lesions, are recorded as non-target lesions. These lesions are not measured individually but are assessed qualitatively. Lesions that are too small to be reliably measured should not be recorded but should be evaluated for progression.

2.3 Evaluation of response in target lesions

The sum of diameters of target lesions serves as the reference for categorising responses:

Complete response (CR): disappearance of all target lesions.

Partial response (PR): at least a 30 % decrease in the sum of diameters of target lesions, taking the baseline sum as the reference.

Progressive disease (PD): at least a 20 % increase in the sum of diameters of target lesions, relative to the smallest sum recorded on study (including baseline), with an absolute increase of at least 5 mm to account for measurement variability, or the appearance of one or more new lesions. Unequivocal progression of non-target

disease or the emergence of new lesions also constitutes PD.

Stable disease (SD): neither sufficient shrinkage to qualify for PR nor sufficient increase to qualify for PD.

Additional considerations:

When multiple lesions show small increases that individually do not reach the 20 % threshold but collectively suggest clear progression, clinical judgement should be used to classify as PD.

Morphological changes such as splitting or coalescence of lesions should be carefully interpreted. For example, if a lesion becomes confluent with another, the combined longest diameter should be used for comparison.

2.3.3 Evaluation of non-target lesions

Non-target lesions include all metastases and lesions that are not selected as target lesions. Their presence or absence contributes to the overall response assessment. At each follow-up visit, non-target lesions are assessed qualitatively:

CR: disappearance of all non-target lesions and normalisation of tumour marker levels.

Non-CR/non-PD: persistence of one or more non-target lesions or abnormal tumour markers without unequivocal progression.

PD: unequivocal progression of existing non-target lesions or the appearance of one or more new lesions. Progression of non-target lesions should be unambiguous; confirmatory evaluation is recommended when feasible.

2.3.4 New lesions

The appearance of one or more new lesions on imaging or pathology constitutes PD. When new lesions are suspected but confirmation is uncertain, therapy should continue and the patient should be re-evaluated at the next scheduled assessment. Positivity on FDG-PET must be confirmed by CT, MRI or histology to differentiate new lesions from inflammatory changes.

2.4 Best overall response (BOR)

The best overall response is the best response recorded from the start of treatment until disease progression or the end of therapy. When a study requires confirmation of CR or PR, response at each time point must satisfy the criteria outlined below. Table 1 and Table 2 summarise the time-point responses for patients with and without target lesions. Table 3 shows how the BOR is derived when confirmation of CR or PR is required.

General rules:

The first assessment of PD determines the BOR as PD, even if subsequent evaluations show non-PD.

SD requires at least one evaluation ≥ 6 weeks after the start of therapy.

Patients without adequate follow-up or who die before assessment should be classified as Not Evaluated (NE).

2.4.1 Time-point response for subjects with target lesions (Table 1)

Target lesions	Non- target lesions	New lesions	Overall response
CR	CR	None	CR
CR	Non- CR/non- PD	None	PR
CR	NE	None	PR
PR	Non- PD or NE	None	PR
SD	Non- PD or NE	None	SD
PD	Any	Any	PD
NE	PD	Any	PD
NE	Non- PD	Any	NE

Abbreviations: CR = complete response; PR = partial response; SD = stable disease; PD = progressive disease; NE = not evaluable.

2.4.2 Time-point response for subjects with only non-target lesions (Table 2)

**Non- target lesions**	**New lesions**	**Overall response**
CR	None	CR
Non- CR/non- PD	None	Non- CR/non- PD
NE	None	NE
PD	Any	PD

2.5 Frequency of tumour re-evaluation

The frequency of tumour assessment should be consistent across study arms and generally every 6–8 weeks. Early confirmation may be necessary for suspected CR or PR.

2.6 Response confirmation

Confirmation of PD is not required. However, confirmation of CR or PR with a repeat assessment at least 4 weeks after the initial response is recommended, particularly in trials where objective response is a primary endpoint.

Appendix 3. Common Terminology Criteria for Adverse Events (CTCAE v5.0)

The table below summarises common adverse events associated with anti-neoplastic therapies and their grading according to the CTCAE v5.0. Grade 1 corresponds to mild, Grade 2 to moderate, Grade 3 to severe or medically significant but not immediately life-threatening, Grade 4 to life-threatening consequences requiring urgent intervention, and Grade 5 to death.

Hematologic system

**Adverse event**	**Grade 1**	**Grade 2**	**Grade 3**	**Grade 4**	**Grade 5**
**White**	Below the	< 3 000 to	< 2 000 to	< 1 000 /mm³	–

Adverse event	Grade 1	Grade 2	Grade 3	Grade 4	Grade 5
**blood cell count decreased**	lower limit of normal (LLN) to 3 000 /mm³	2 000 /mm³	1 000 /mm³		
**Neutrophil count decreased**	< LLN to 1 500 /mm³	< 1 500 to 1 000 /mm³	< 1 000 to 500 /mm³	< 500 /mm³	–
**Platelet count decreased**	< LLN to 75 000 /mm³	< 75 000 to 50 000 /mm³	< 50 000 to 25 000 /mm³	< 25 000 /mm³	–
**Anaemia**	Haemoglobin < LLN to 10.0 g/dL	Haemoglobin < 10.0 to 8.0 g/dL	Haemoglobin < 8.0 g/dL; transfusion indicated	Life- threatening; urgent intervention indicated	Death

Hepatic and gastrointestinal

Adverse event	Grade 1	Grade 2	Grade 3	Grade 4	Grade 5
**Total bilirubin increased**	> ULN to 1.5 × ULN	> 1.5 to 3.0 × ULN	> 3.0 to 10.0 × ULN	> 10.0 × ULN	–

**Alanine aminotransferase (ALT) increased**	> ULN to 3.0 × ULN	> 3.0 to 5.0 × ULN	> 5.0 to 20.0 × ULN	> 20.0 × ULN	–
**Aspartate aminotransferase (AST) increased**	> ULN to 3.0 × ULN	> 3.0 to 5.0 × ULN	> 5.0 to 20.0 × ULN	> 20.0 × ULN	–
**Alkaline phosphatase increased**	> ULN to 2.5 × ULN	> 2.5 to 5.0 × ULN	> 5.0 to 20.0 × ULN	> 20.0 × ULN	–
**Nausea**	Decreased appetite without change in eating habits	Reduced oral intake without significant weight loss, dehydration or malnutrition	Inadequate oral caloric or fluid intake; tube feeding, total parenteral nutrition or hospitalisation required	–	–
**Vomiting**	No intervention	Outpatient intravenous	Tube feeding, total	Life- threatening; urgent	Death

	s required	fluids or medical intervention required	parenteral nutrition or hospitalisation required	intervention required	
**Diarrhoea**	< 4 stools per day over baseline; mild increase in ostomy output	4–6 stools per day over baseline or moderate increase in ostomy output; limitation in instrumental activities of daily living (ADL)	≥ 7 stools per day over baseline; incontinence; hospitalisation indicated; severe increase in ostomy output; limitation in self- care ADL	Life- threatening; urgent intervention required	Death

Kidney and bladder

**Adverse event**	**Grade 1**	**Grade 2**	**Grade 3**	**Grade 4**	**Grade 5**

**Acute kidney injury**	–	–	Hospitalisation indicated	Life- threatening; dialysis required	Death
**Haematuria**	Asymptomatic; detected only by clinical observation or diagnostics; no intervention required	Symptomatic; catheterisation or bladder irrigation required; limitation in instrumental ADL	Gross haematuria requiring transfusion, intravenous medications or hospitalisation; elective invasive intervention indicated; limitation in self- care ADL	Life- threatening; emergency invasive intervention required	Death

**Proteinuria**	1+ proteinuria; 24- hour urine protein ≥ ULN but < 1.0 g	For adults: 2+–3+ proteinuria; 24- hour urine protein 1.0–3.5 g; for children: urine protein/creatinine ratio 0.5–1.9	For adults: 24- hour urine protein ≥ 3.5 g or 4+ proteinuria; for children: urine protein/creatinine ratio > 1.9	–	–

Cardiac disorders

**Adverse event**	**Grade 1**	**Grade 2**	**Grade 3**	**Grade 4**	**Grade 5**
**Left ventricular systolic dysfunction**	–	–	Decrease in ejection fraction associated with symptoms, responsive to treatment	Marked reduction in ejection fraction with refractory or poorly controlled heart failure; need for ventricular assist device,	**Death**

				intravenous inotropic therapy or heart transplantation	
**Myocardial infarction**	–	Asymptomatic; minor cardiac enzyme changes without ischaemic ECG changes	Severe symptoms with cardiac enzyme elevation; haemodynamically stable; ECG changes consistent with myocardial infarction	Life- threatening; haemodynamic instability	**Death**

**Heart failure**	Asymptomatic; abnormal findings on laboratory (e.g. natriuretic peptides) or cardiac imaging	Symptomatic with moderate activity or exertion; new symptoms requiring hospitalisation	Severe symptoms at rest or with minimal activity; hospitalisation indicated; need for continuous infusion or mechanical circulatory support	Life- threatening; urgent intervention required	**Death**

Other

**Adverse event**	**Grade 1**	**Grade 2**	**Grade 3**	**Grade 4**	**Grade 5**
**Constipation**	Intermittent or occasional; occasional use of stool softeners, laxatives, dietary	Persistent use of laxatives or enemas; limitation in instrumental ADL	Obstinate constipation requiring manual disimpaction; limitation in self- care ADL	Life- threatening; urgent intervention required	Death

	adjustment or enemas				
**Pain**	Mild pain	Moderate pain limiting routine household activities	Severe pain limiting self- care ADL	–	–
**Allergic reaction**	–	–	Symptomatic bronchospasm with or without urticaria; requires parenteral therapy; angio- oedema/oedema associated with hypersensitivity reactions; hypotension	Life- threatening; urgent intervention required	Death
**Fever**	38.0–39.0 °C	> 39.0–40.0 °C	> 40.0 °C, ≤ 24 hours	> 40.0 °C for > 24 hours	Death
**Alopecia**	Hair loss affecting < 50 % of	Hair loss affecting > 50 % of scalp;	–	–	–

	scalp; not obvious at a distance but visible upon close inspection	obvious; requires wig or hairpiece for complete coverage; may be associated with psychological impact			
