# Supplementary material for: CCL3+ Neutrophil Signature Predicts Response to Neoadjuvant Toripalimab plus Chemotherapy in Patients with Hypopharyngeal Squamous Cell Carcinoma: A Phase II Trial
Source: Clin Cancer Res. 2026 Mar 12;32(11):2166–82. doi: 10.1158/1078-0432.CCR-25-4096 (PMC13223550; doi:10.1158/1078-0432.CCR-25-4096)
Supplement: Supplementary Figure S1 — Comparison of clinical outcomes and survival analyses across different T/N stages and PD-L1 CPS status. [file ccr-25-4096_supplementary_figure_s1_suppfs1.pdf]

Supplementary Figure S1

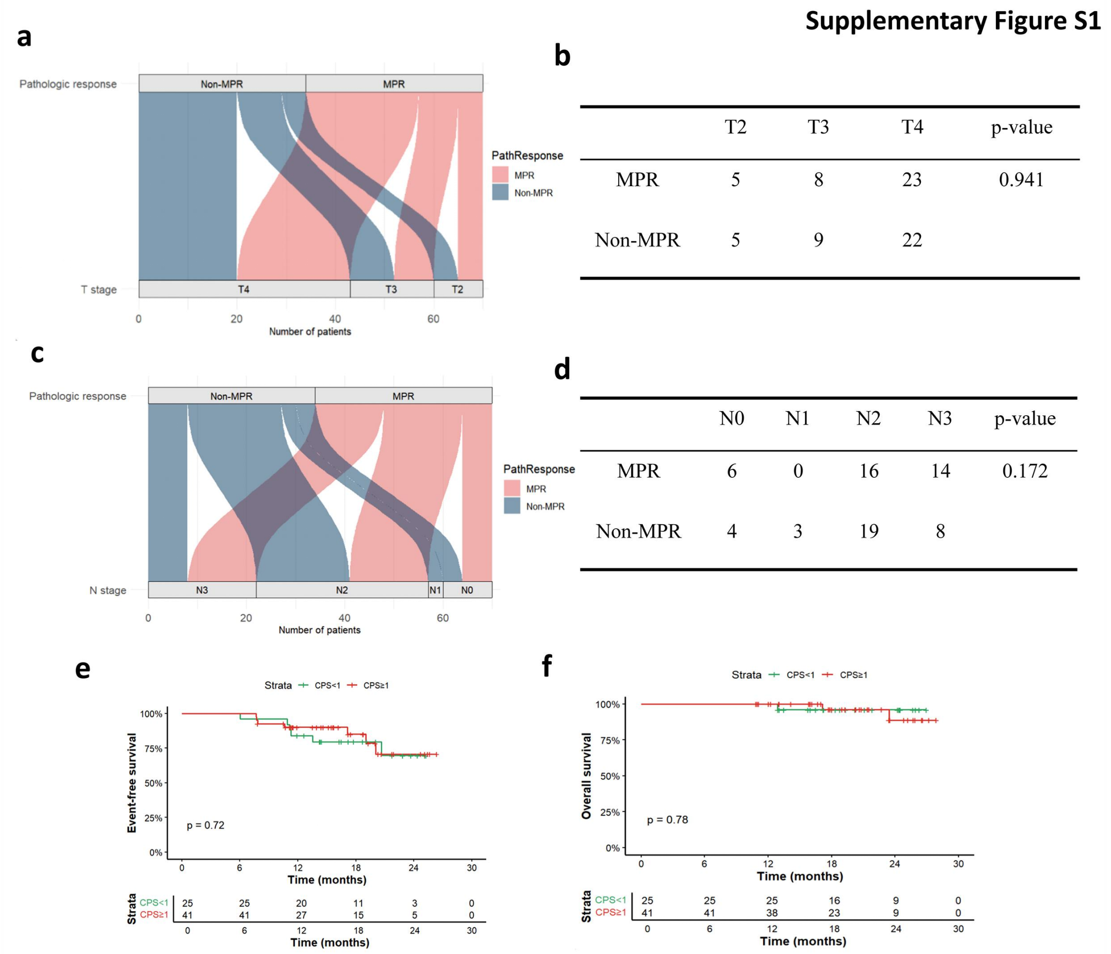

b

	T2	T3	T4	p-value
MPR	5	8	23	0.941
Non-MPR	5	9	22	

d

	N0	N1	N2	N3	p-value
MPR	6	0	16	14	0.172
Non-MPR	4	3	19	8	

Supplementary Figure S1: Comparison of clinical outcomes and survival analyses across different T/N stages and PD-L1 CPS status.

(a) Sankey diagram showing the concordance between T stage and pathological response category (MPR vs non-MPR) in 64 patients who underwent surgery. (b) Contingency table analysis indicates no significant difference in MPR rates among patients with different T stages (two-sided Fisher's exact test). (c) Sankey diagram showing the concordance between N stage and pathological response category (MPR vs non-MPR) in 64 surgical patients. (d) Contingency table analysis indicates no significant difference in MPR rates among patients with different N stages (two-sided Fisher's exact test). (e) Kaplan–Meier overall survival curves stratified by

pre-treatment PD-L1 CPS status (CPS ≥ 1 vs CPS < 1, n = 66). (f) Kaplan–Meier event-free survival curves stratified by pre-treatment PD-L1 CPS status (CPS ≥ 1 vs CPS < 1, n = 66).
