# Supplementary material for: CCL3+ Neutrophil Signature Predicts Response to Neoadjuvant Toripalimab plus Chemotherapy in Patients with Hypopharyngeal Squamous Cell Carcinoma: A Phase II Trial
Source: Clin Cancer Res. 2026 Mar 12;32(11):2166–82. doi: 10.1158/1078-0432.CCR-25-4096 (PMC13223550; doi:10.1158/1078-0432.CCR-25-4096)
Supplement: Supplementary Figure S2 — Clinical overview, major lineage annotation, and differential expression analyses of the scRNA-seq discovery cohort. [file ccr-25-4096_supplementary_figure_s2_suppfs2.pdf]

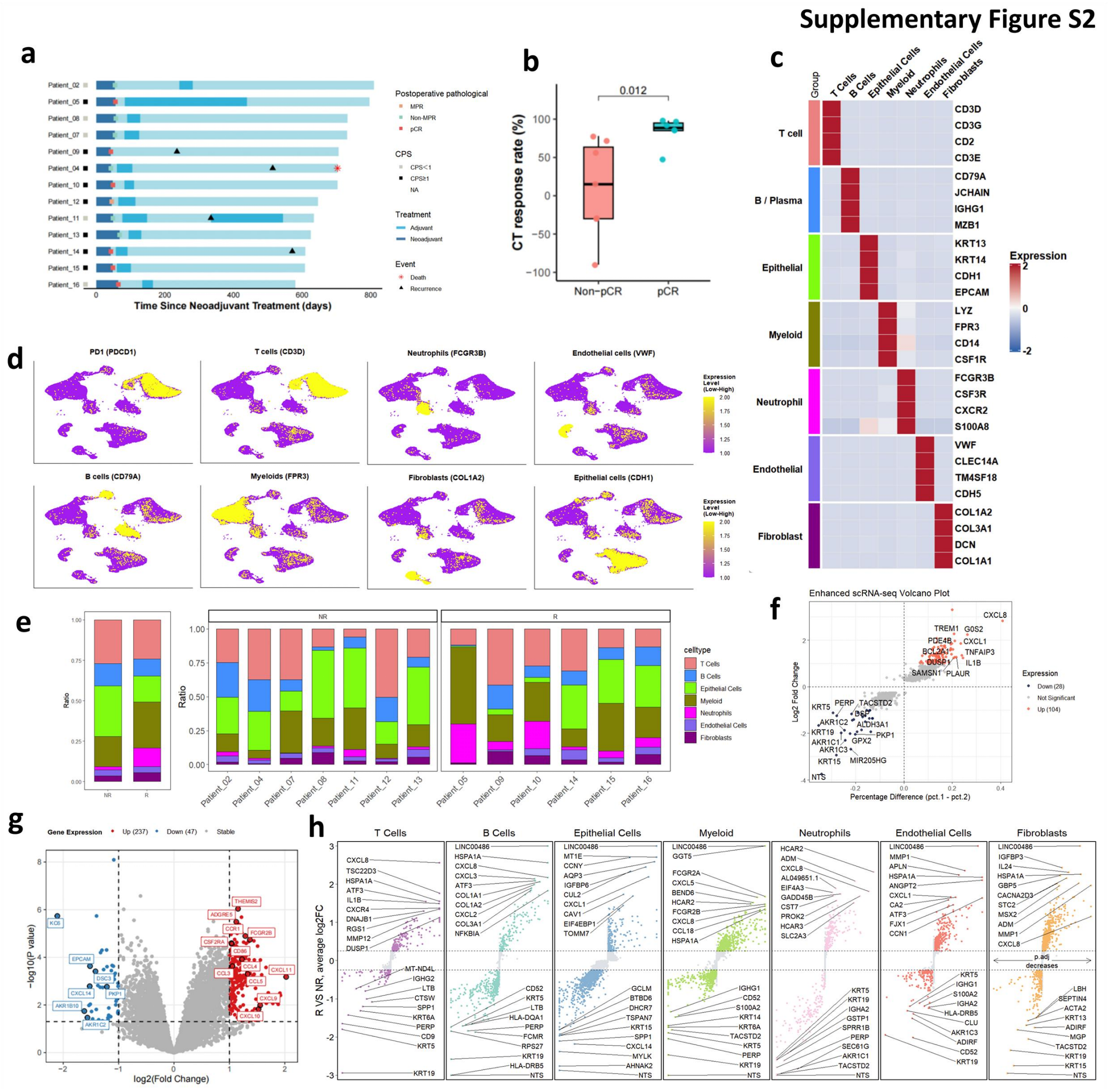

Supplementary Figure S2: Clinical overview, major lineage annotation, and differential expression analyses of the scRNA-seq discovery cohort.

(a) Swimmer plot summarizing the clinical course of the 13 patients profiled by scRNA-seq, annotated by postoperative pathological response, CPS, treatment, and clinical events (recurrence and death), with time shown as days since initiation of neoadjuvant treatment. (b) Radiologic response rate assessed by CT in the same cohort, stratified by pCR versus non-pCR. (c) Heatmap showing scaled expression of representative marker genes used to define major cell lineages (T cells, B/plasma cells, epithelial cells, myeloid cells, neutrophils, endothelial cells, and fibroblasts) from the initial clustering. (d) UMAP feature plots displaying the expression of canonical lineage

markers (PDCD1, CD3D, FCGR3B, VWF, CD79A, FPR3, COL1A2, and CDH1) supporting major lineage annotation. (e) Stacked bar plots showing the relative proportions of major cell lineages across response groups and across individual patient samples. (f) Differential expression summary from the enhanced scRNA-seq analysis comparing responders (R) and non-responders (NR), with representative genes highlighted. (g) Volcano plot showing differentially expressed genes between R and NR at the cohort level. (h) Cell type–resolved differential expression comparing R versus NR within each major lineage, with representative genes labeled.
