# Supplementary material for: CCL3+ Neutrophil Signature Predicts Response to Neoadjuvant Toripalimab plus Chemotherapy in Patients with Hypopharyngeal Squamous Cell Carcinoma: A Phase II Trial
Source: Clin Cancer Res. 2026 Mar 12;32(11):2166–82. doi: 10.1158/1078-0432.CCR-25-4096 (PMC13223550; doi:10.1158/1078-0432.CCR-25-4096)
Supplement: Supplementary Figure S3 — Epithelial CNV/stemness features, epithelial–neutrophil communication, and immune-cell states associated with response. [file ccr-25-4096_supplementary_figure_s3_suppfs3.pdf]

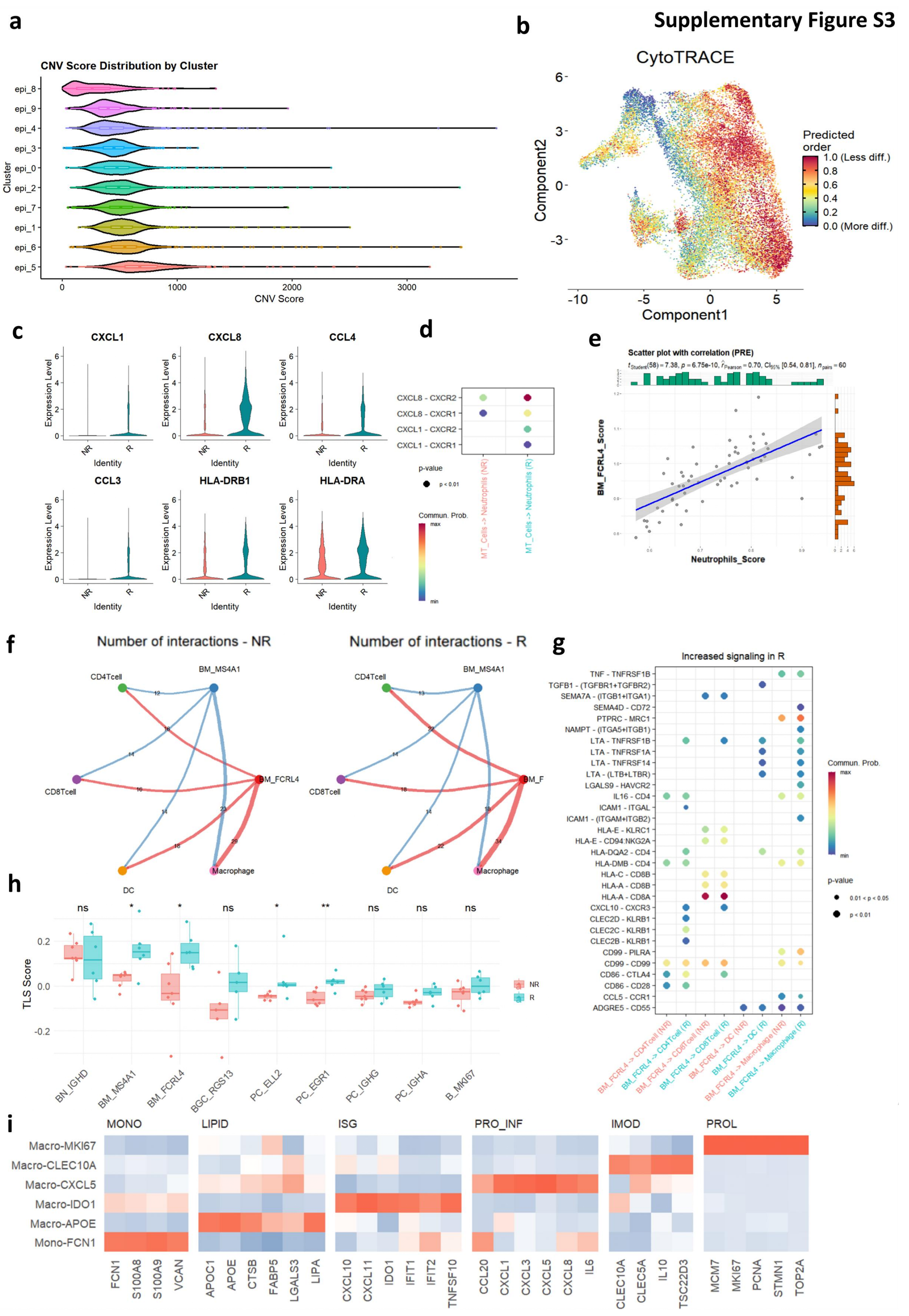

Supplementary Figure S3: Epithelial CNV/stemness features, epithelial–neutrophil communication, and immune-cell states associated with response.

(a) Violin plots showing inferred CNV scores across epithelial clusters. (b) UMAP of epithelial cells colored by CytoTRACE score (higher scores indicate a less-differentiated, more progenitor-like state). (c) Violin plots comparing expression of selected chemokines and antigen-presentation genes (CXCL1, CXCL8, CCL4, CCL3, HLA-DRA, HLA-DRB1) in MT epithelial cells between responders (R) and non-responders (NR). (d) CellChat dot plot summarizing chemokine ligand–receptor interactions from MT epithelial cells (sender) to neutrophils (receiver) in R versus NR; dot color indicates communication probability and dot size reflects significance. (e) Scatter plot with marginal histograms showing the correlation between the BM_FCRL4 signature score and the neutrophil signature score in the bulk RNA-seq cohort (pre-treatment), with correlation statistics shown. (f) CellChat network plots comparing the number of significant ligand–receptor interactions from B-cell subsets to other immune populations in NR versus R. (g) CellChat dot plot highlighting ligand–receptor interactions increased in R in which BM_FCRL4 cells act as the sender; color indicates communication probability and dot size indicates significance. (h) Box plots comparing tertiary lymphoid structure (TLS) signature scores across B-cell subsets between NR and R; ns, not significant; *$P < 0.05$; **$P < 0.01$. (i) Heatmap showing scaled expression of representative marker genes defining macrophage/monocyte programs across myeloid subsets (e.g., MONO, LIPID, ISG, PRO-INF, IMOD, and PROL).
