# Supplementary material for: CCL3+ Neutrophil Signature Predicts Response to Neoadjuvant Toripalimab plus Chemotherapy in Patients with Hypopharyngeal Squamous Cell Carcinoma: A Phase II Trial
Source: Clin Cancer Res. 2026 Mar 12;32(11):2166–82. doi: 10.1158/1078-0432.CCR-25-4096 (PMC13223550; doi:10.1158/1078-0432.CCR-25-4096)
Supplement: Supplementary Figure S4 — Validation of Neu_CCL3 in pan-cancer nCIT cohorts. [file ccr-25-4096_supplementary_figure_s4_suppfs4.pdf]

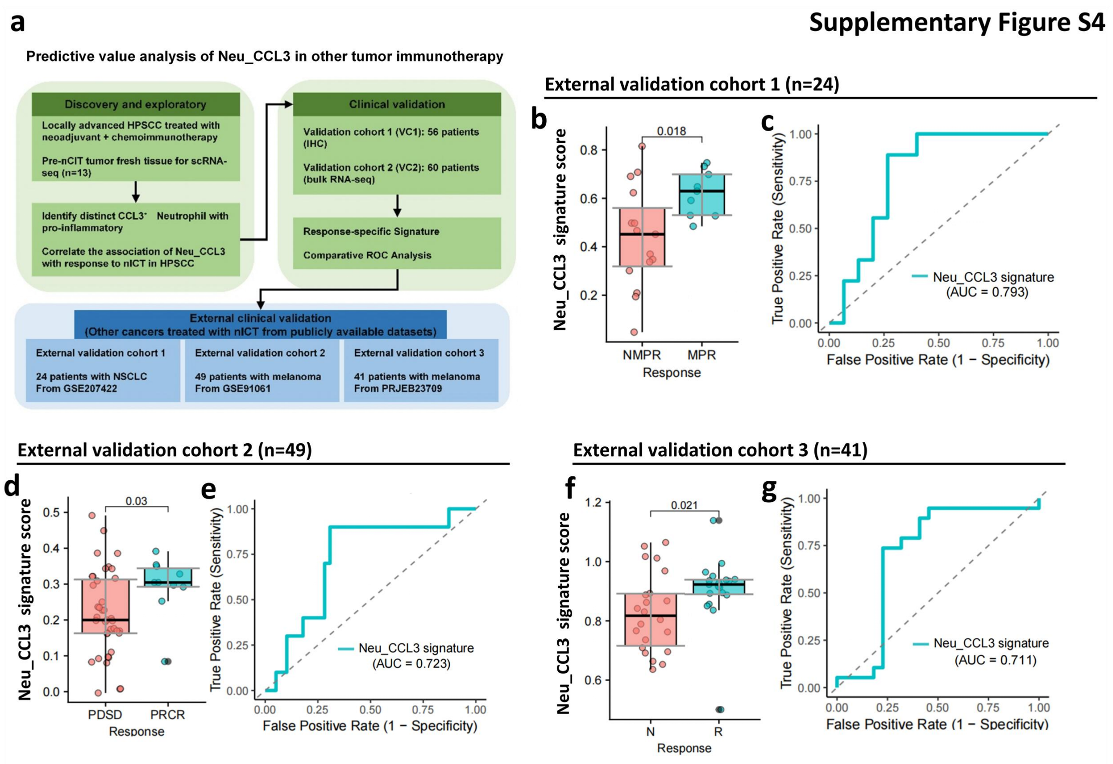

Supplementary Figure S4: Validation of Neu_CCL3 in pan-cancer nCIT cohorts.

(a) Study design and validation workflow across the discovery/exploratory cohort, clinical validation cohorts, and external validation cohorts. (b–g) Association of the Neu_CCL3 signature with treatment response in external nCIT cohorts. External cohort 1: NMPR vs MPR. External cohort 2: PD/SD vs PR/CR (PD, progressive disease; SD, stable disease; PR, partial response; CR, complete response). External cohort 3 (PRJEB23709): N, non-responder; R, responder (as annotated in the original dataset).
