# Supplementary material for: CCL3+ Neutrophil Signature Predicts Response to Neoadjuvant Toripalimab plus Chemotherapy in Patients with Hypopharyngeal Squamous Cell Carcinoma: A Phase II Trial
Source: Clin Cancer Res. 2026 Mar 12;32(11):2166–82. doi: 10.1158/1078-0432.CCR-25-4096 (PMC13223550; doi:10.1158/1078-0432.CCR-25-4096)
Supplement: Supplementary Figure S5 — Anti-Ly6G depletion and tumor weights [file ccr-25-4096_supplementary_figure_s5_suppfs5.pdf]

Supplementary Figure S5

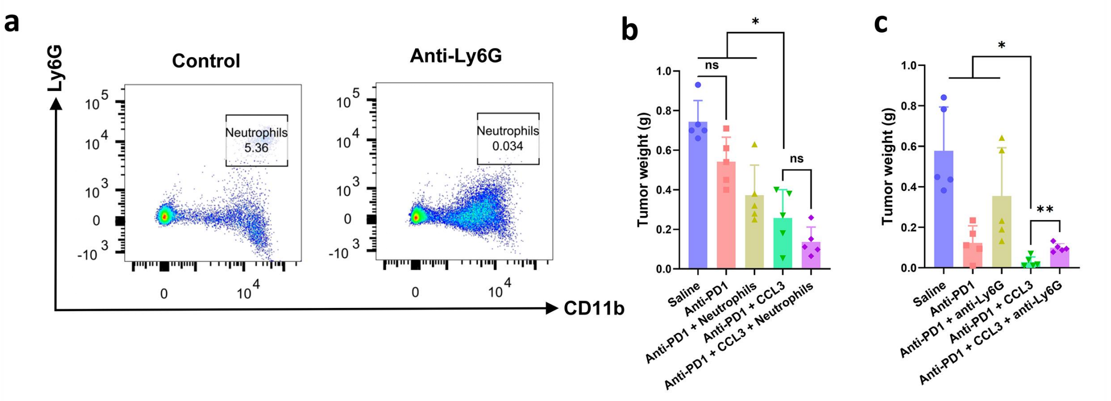

Supplementary Figure S5: Anti-Ly6G depletion and tumor weights

(a) Representative flow-cytometry plots (CD11b vs Ly6G) showing efficient depletion of neutrophils after anti-Ly6G treatment compared with control. (b) Endpoint tumor weights in mice treated with saline or anti–PD-1, with additional neutrophil transfer and/or CCL3 as indicated. (c) Endpoint tumor weights in mice treated with anti–PD-1 ± anti-Ly6G, with or without CCL3 as indicated. Data are shown as mean ± SD (n = 5 mice per group). Two-sided t-test for the comparisons indicated. ns, not significant; * $p < 0.05$; ** $p < 0.01$.
