# Supplementary material for: CCL3+ Neutrophil Signature Predicts Response to Neoadjuvant Toripalimab plus Chemotherapy in Patients with Hypopharyngeal Squamous Cell Carcinoma: A Phase II Trial
Source: Clin Cancer Res. 2026 Mar 12;32(11):2166–82. doi: 10.1158/1078-0432.CCR-25-4096 (PMC13223550; doi:10.1158/1078-0432.CCR-25-4096)
Supplement: Supplementary Figure S6 — Functional characterization of T-cell states and lymphocyte-activity signatures in responders versus non-responders. [file ccr-25-4096_supplementary_figure_s6_suppfs6.pdf]

Supplementary Figure S6

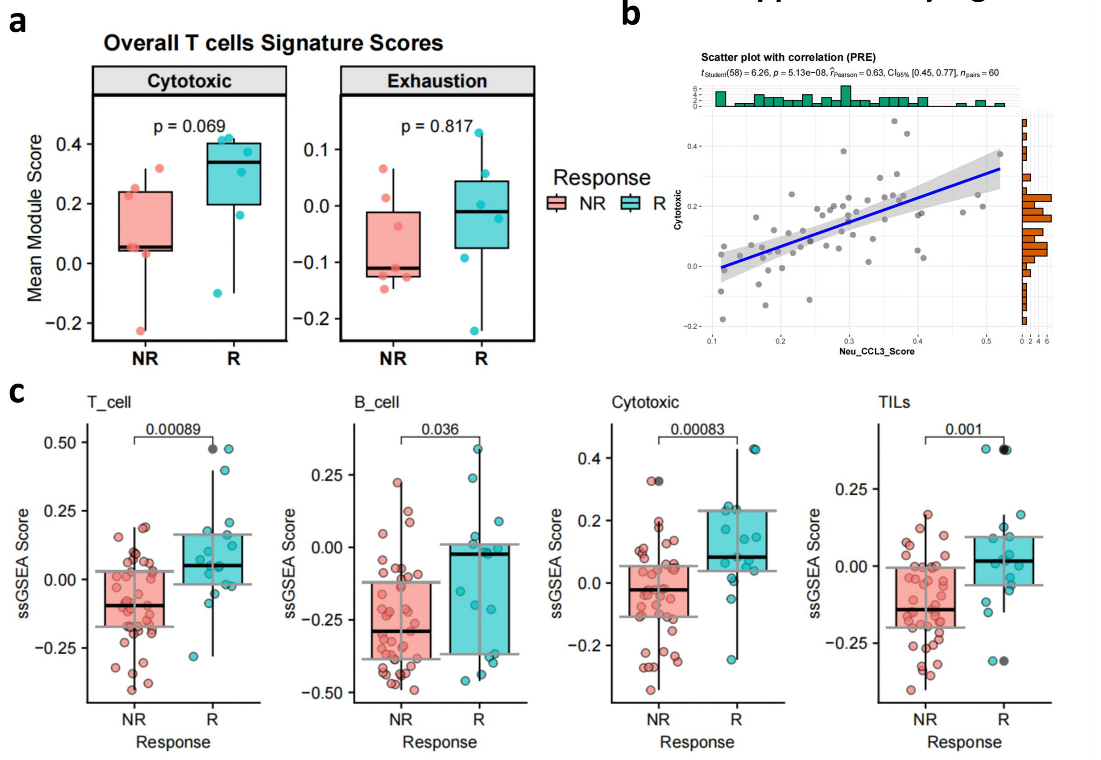

Supplementary Figure S6. Functional characterization of T-cell states and lymphocyte-activity signatures in responders versus non-responders.

(a) cytotoxic & exhaustion module scores in scRNA T cells. (b) correlation between Neu_CCL3 score and cytotoxic signature in bulk. (c) ssGSEA scores (T cell, B cell, cytotoxic, T cell–inflamed GEP).
