# Supplementary material for: CCL3+ Neutrophil Signature Predicts Response to Neoadjuvant Toripalimab plus Chemotherapy in Patients with Hypopharyngeal Squamous Cell Carcinoma: A Phase II Trial
Source: Clin Cancer Res. 2026 Mar 12;32(11):2166–82. doi: 10.1158/1078-0432.CCR-25-4096 (PMC13223550; doi:10.1158/1078-0432.CCR-25-4096)
Supplement: Supplementary Table S1 — Representativeness of Study Participants [file ccr-25-4096_supplementary_table_s1_suppts1.pdf]

Supplementary Table S1. Representativeness of Study Participants

Cancer type(s)/subtype(s)/stage(s)/condition	Hypopharyngeal squamous cell carcinoma (HPSCC)
Considerations related to:	
Sex	HPSCC shows marked male predominance in population-based series, with approximately 80%–90% of cases occurring in men, reflecting differences in tobacco and alcohol exposure.
Age	HPSCC primarily affects older adults; the typical median age at diagnosis is in the early-to-mid 60s, and most cases occur in individuals ≥50 years, with incidence increasing with age.
Race/ethnicity	Incidence varies geographically. In China, most clinical cohorts are ethnically homogeneous (predominantly Han), which limits assessment of race/ethnicity-related disparities within a single-center cohort.<br>Accordingly, race/ethnicity-related disparities cannot be assessed within a single-center Chinese cohort, which may limit generalizability to more diverse populations.
Geography	As a referral surgical center, enrolled patients may over-represent locally advanced, surgically manageable disease and those with access to specialized multimodal care.
Other considerations	Tobacco use and alcohol consumption are common etiologic risk factors. Many patients present with advanced T category and/or nodal disease, and tumors arise most commonly in the pyriform sinus. Differences in comorbidity profiles, socioeconomic status, and access to care can influence enrollment in clinical studies. Unlike oropharyngeal cancer, HPV-associated disease is less prominent in HPSCC, and traditional exposures (tobacco/alcohol) remain dominant risk factors in most Chinese cohorts.
Overall representativeness of this study	Our cohort is representative of clinic-based, locally advanced HPSCC populations in China. All participants were male, consistent with the strong male predominance of HPSCC. The median age was 63 years (range, 42 – 75),

	aligning with the typical population-level median (~60 – 65 years). Smoking and alcohol exposure were highly prevalent, in keeping with established etiologic risk factors. Most patients presented with locally advanced disease and frequent nodal involvement, which is typical of referral-center HPSCC. However, the single-center design and ethnically homogeneous population limit generalizability to female patients and more diverse geographic/ethnic settings.
