# Supplementary material for: CCL3+ Neutrophil Signature Predicts Response to Neoadjuvant Toripalimab plus Chemotherapy in Patients with Hypopharyngeal Squamous Cell Carcinoma: A Phase II Trial
Source: Clin Cancer Res. 2026 Mar 12;32(11):2166–82. doi: 10.1158/1078-0432.CCR-25-4096 (PMC13223550; doi:10.1158/1078-0432.CCR-25-4096)
Supplement: Supplementary Table S2 — Key Resources Table [file ccr-25-4096_supplementary_table_s2_suppts2.pdf]

Supplementary Table S2. Key Resources Table

**Reagent or Resource**	**Source**	**Identifier**
**Antibodies**		
Rabbit anti-human CD15	Abcam	Cat# ab135377; RRID:AB_3662850
Rabbit anti-human CD68	Abcam	Cat# ab303565; RRID:AB_3075482
Rabbit anti-human CD15	Abcam	Cat# ab241552; RRID:AB_3675595
Rabbit anti-human CCL3	Abcam	Cat# ab259372; RRID:AB_3094596
Rat IgG2a isotype control	Selleck	Cat# A2123; RRID:AB_3644245
Anti-mouse Ly6G	Selleck	Cat# A2158; RRID:AB_3677246
Anti-mouse PD-1	BioXCell	Cat# BE0146; RRID:AB_10949053
Anti-CD45 (Fluorochrome-conjugated)	Thermo Fisher Scientific	Cat# 12-0451-82; RRID:AB_465668
Anti-CD3 (Fluorochrome-conjugated)	Thermo Fisher Scientific	Cat# 11-0032-82; RRID:AB_2572431
Anti-CD8a (Fluorochrome-conjugated)	Thermo Fisher Scientific	Cat# 17-0081-82; RRID:AB_469335
Anti-CD11b (Fluorochrome-conjugated)	Thermo Fisher Scientific	Cat# 11-0112-41; RRID:AB_11042156
Anti-Ly6G (Fluorochrome-conjugated)	Thermo Fisher Scientific	Cat# 47-9668-82; RRID:AB_2802291
**Experimental Models: Cell Lines**		
Mouse: MOC1	Qingqi (Shanghai) Biotechnology	RRID:CVCL_ZD32
Mouse: MOC2	Qingqi (Shanghai) Biotechnology	RRID:CVCL_ZD33

**Experimental Models: Organisms/Strains**		
Mouse: C57BL/6J	Animal Center of Shandong University	RRID:IMSR_JAX:000664
**Software and Algorithms**		
R Project for Statistical Computing (v4.3.1)	R Project	RRID:SCR_001905
Seurat (v5.0.3)	Satija Lab	RRID:SCR_016341
Harmony (v1.2.0)	Korsunsky Lab	RRID:SCR_022206
CellChat (v1.6.1)	Jin Lab	RRID:SCR_021946
inferCNV (v1.18.1)	Broad Institute	RRID:SCR_021140
fgsea (v1.28.0)	Bioconductor	RRID:SCR_020938
msigdbr (v7.5.1)	CRAN	RRID:SCR_022870
STAR aligner (v2.7.10b)	Dobin et al.	RRID:SCR_004463
HTSeq-count (v0.13.5)	Anders et al.	RRID:SCR_005514
QuPath	Bankhead et al.	RRID:SCR_018257
pROC (v1.18.5)	Robin et al.	RRID:SCR_024286
