# Supplementary material for: CCL3+ Neutrophil Signature Predicts Response to Neoadjuvant Toripalimab plus Chemotherapy in Patients with Hypopharyngeal Squamous Cell Carcinoma: A Phase II Trial
Source: Clin Cancer Res. 2026 Mar 12;32(11):2166–82. doi: 10.1158/1078-0432.CCR-25-4096 (PMC13223550; doi:10.1158/1078-0432.CCR-25-4096)
Supplement: Supplementary Table S3 — Patient characteristics [file ccr-25-4096_supplementary_table_s3_suppts3.pdf]

Supplementary Table S3. Patient characteristics

Variables	N=70	Percent (%)
**Median age, years (range)**	62(42-75)	-
**Sex, n（%）**		
Male	70	100
**Smoking, n（%）**		
Yes	62	88.5
No	8	11.5
**Alcohol consumption status, n（%）**		
Ever	67	95.7
Never	3	4.3
**Tumor site, n（%）**		
Pyriform sinus	52	74.3
Postcricoid	7	10.0
Posterior hypoharyngeal wall	11	15.7
**T stages 8th, n（%）**		
T1	1	1.4
T2	8	11.4
T3	17	24.3
T4	44	62.9
**N stages 8th, n（%）**		

N0	12	17.1
N1	2	2.9
N2	34	48.6
N3	22	31.4
**CPS, n（%）**		
<1	25	35.7
≥1	40	57.1
Not available	5	7.2

Values are n (%) unless otherwise indicated. Percentages are calculated based on N=70.
