# Supplementary material for: CCL3+ Neutrophil Signature Predicts Response to Neoadjuvant Toripalimab plus Chemotherapy in Patients with Hypopharyngeal Squamous Cell Carcinoma: A Phase II Trial
Source: Clin Cancer Res. 2026 Mar 12;32(11):2166–82. doi: 10.1158/1078-0432.CCR-25-4096 (PMC13223550; doi:10.1158/1078-0432.CCR-25-4096)
Supplement: Supplementary Table S4 — Treatment-related adverse events by grade (N=70) [file ccr-25-4096_supplementary_table_s4_suppts4.pdf]

Supplementary Table S4. Treatment-related adverse events by grade (N=70)

	Grade 1-2		Grade 3		Grade 4	
	**No**	**%**	**No**	**%**	**No**	**%**
Alopecia	70	100.0				
Pneumonia	6	8.6	3	4.3		
Fever	2	2.9				
Rash	6	8.6				
Diarrhea	3	4.3				
Adrenal insufficiency	4	5.7				
Arthritis	2	2.9				
Arrhythmia	1	1.4				
Leukopenia	3	4.3				

Data are number of patients with at least one event, n (%). Percentages are based on N=70. TRAEs were assessed by investigators and graded according to CTCAE v5.0. For patients with multiple episodes of the same event, the maximum grade is reported.
